# Visual opponent mechanisms and spectral responses in non-primate vertebrates: taxonomic distribution, sampling, and classification

**DOI:** 10.7717/peerj.20959

**Published:** 2026-03-20

**Authors:** Carlay L. Teed, Esteban Fernández-Juricic

**Affiliations:** Department of Biological Sciences, Purdue University, West Lafayette, IN, United States of America

**Keywords:** Color vision, Retinal mechanisms, Comparative physiology, Photoreceptors, Neuroethology, Opponency, Horizontal cells, Retinal Ganglion cells, Vision, Sensory systems

## Abstract

Understanding neural mechanisms of opponency is the next frontier of comparative color vision research. Opponent cell properties can be investigated from different perspectives: the type of cone inputs to a cell (cone opponency), and the wavelengths of light to which the cell responds (spectral opponency). Based on a recent database (DOI 10.1016/j.dib.2024.111166) on cone and spectral opponency across non-primate vertebrates, we identified major taxonomic trends in the distribution of opponent cells through image-forming brain regions, analyzed sampling metrics and experimental variables influencing their detection, and established the limitations of existing classification systems. Evidence of opponency is reported in mammals, reptiles (exclusively turtles), amphibians, fish, and birds. While single cones drive every cone opponent cell described, double/twin cones and rods can also contribute in all groups, but reptiles. From a sampling perspective, only 58% of studies reported the number of cells sampled to find cone opponent cells. There are no estimates of the relative abundance of opponent cells, and our best approximation (frequency of opponent cell encounters) may be heavily biased by experimental goals, design, and equipment. To address cross-taxa ambiguity in opponency classification, we developed three classification frameworks for opponent cell types based on the relative positions of photoreceptor peak sensitivities and null point(s). Transparent sampling and universal classification methods will be critical for making evolutionary and functional inferences about opponency in vertebrate vision.

## Introduction

The visual system’s primary function is to detect objects of interest in the environment. Every object reflects a subset of wavelengths in the visible spectrum which the brain interprets as color. These colors can be used to detect and distinguish between objects (Bramão et al., 2011; Regan et al., 2001). To detect objects of different colors, the retina possesses photoreceptors that are sensitive to different portions of the visible spectrum. Different classes of photoreceptors produce different signals in response to the same color of light (Fig. S1A). The signals from these photoreceptors are compared by neurons in the brain to determine the color of objects (Daw, 2009; Gouras, 2009). The best understood method of photoreceptor signal comparison is the antagonistic comparison known as opponency (Daw, 1973; Thoreson & Dacey, 2019) (Fig. S1Aii). We can approach the study of opponency from three perspectives: (1) the neural components involved (cone opponency); (2) how opponent cells respond to light (spectral opponency); and (3) how opponent cells contribute to the perception of color (color opponency).

The study of the biological mechanisms of opponent cells has been ongoing since the 1950s. Recently, Teed, Hartzler & Fernández-Juricic (2024) compiled these studies across non-primate vertebrate species. Teed, Hartzler & Fernández-Juricic (2024) did not include primates in their dataset as their opponent mechanisms have been explored elsewhere (Gouras & Zrenner, 1981; Jayakumar, Dreher & Vidyasagar, 2013). In this review, we synthesize the literature compiled in the Teed, Hartzler & Fernández-Juricic (2024) dataset, striving to answer the following questions, which are each addressed in separate sections:

(1) Which taxa and regions of the visual system are known to use cone and spectral opponency to process color signals?
(2) Is the sampling effort applied in these studies sufficient to describe the diversity of existing cone opponent cell types?
(3) How should one classify opponent cells for interspecies comparisons?

Our first section, “Distribution of Opponency Across Species”, addresses question one by providing an overview of the taxonomic representation of studies that characterized cone and spectral opponency, highlighting trends within major vertebrate classes and between different layers of the retina. In our second section, “Sampling Opponent Cells”, we extracted sample size metrics from different studies and addressed the sampling effort required to find opponent cell types in different visual system layers and major vertebrate classes. In our third section, “Opponent Cell Classifications”, we explore the existing classifications used to distinguish between opponent cell types. These classifications reference either the subjective color (*e.g.*, red) a cell responds to, or the specific photoreceptors (*e.g.*, “L”) that the cell is connected to. When answering question 3, we found that existing classifications introduce ambiguity when trying to draw comparisons across taxa. Therefore, we developed a standardized cell type classification that can be used for comparative analyses.

Overall, this review identifies critical gaps in our knowledge, offers a unified classification system that can be used to conduct comparative analyses, and synthesizes existing information for the design of future studies on cone and spectral opponency. Consequently, we believe this contribution is of direct relevance to researchers and students in ecology and neuroscience, where understanding sensory processing shapes interpretations of animal behavior, evolution, and neural circuitry. We also included a background section which breaks down the fundamentals of opponency, starting with signal transduction from different types of photoreceptors, making this piece an inviting resource for non-specialists and interdisciplinary scholars seeking an entry point into the study of vertebrate color vision mechanisms.

## Survey Methodology

This project consisted of two steps: (1) the publication of the raw information on opponency collated from the literature as a data paper (see Teed, Hartzler & Fernández-Juricic (2024), DOI: http://dx.doi.org/10.1016/j.dib.2024.111166), and (2) the present review of that literature. To collect the data, we conducted a reproducible literature search following PRISMA guidelines (Page et al., 2021). After exploring the literature independently, we developed search terms to query Web of Science and Scopus with the help of a librarian (Fig. S2). This initial search identified 1,398 manuscripts to review. When reviewing titles and abstracts, we excluded manuscripts on the following topics: non-visual senses and organs, visual phenomena unrelated to color vision, injury or disease, evolution and behavior, and molecular biology or genetics. When reading the manuscripts to determine which to accept, we excluded papers on the following topics: behavior and evolution, disease, non-visual organs and senses, genetics, retinal development, or molecular biology. We also excluded studies that did not include any electrophysiology or did not specify the species studied (*e.g.*, only identifying the species as a “goldfish” without specifying *Carassius auratus vs Carassius carassius)*. Additionally, we had a strict acceptance criterion; we only accepted papers that provided a complete description of at least one type of opponent cell. For example, we did not accept studies only describing the excitatory phase and neglecting the inhibitory phase. Nor did we accept cone opponency studies that were uncertain of the cone types present in the species. After reviewing titles, abstracts, and the manuscripts themselves, we accepted 73 manuscripts.

Since we noticed that some key papers from our initial exploration were not revealed in our first search, we performed one round of citation chasing based on all accepted papers. From this round of citation chasing we identified 2,133 additional manuscripts to review, from which we included 47 more manuscripts (Fig. S2). In total, we evaluated 5,208 manuscripts from Web of Science and Scopus and ultimately included 120 manuscripts written in English, Greek, Japanese, German, Korean, and Russian. More information on our search process can be found at Teed, Hartzler & Fernández-Juricic (2024).

For this review, the data from the Teed, Hartzler & Fernández-Juricic (2024) dataset was further processed to make data within a species comparable across studies. We found occasional mismatches between the cones reported to contribute to opponency, and the photoreceptor compliment reported to exist in a species. For example, some zebrafish (*Danio rerio*) studies report that opponent cells which respond to red and green light are driven by L and M cones (*e.g.*, Zimmermann et al., 2018). However, according to photoreceptor studies, zebrafish do not have L and M cones; instead, these responses are driven by input from each member of the double cone (Cameron, 2002). Additionally, we found that studies on the same species, by different authors, reported different photoreceptor compliments within the species. In the mouse (*Mus musculus*) for example, some studies name the two cones which mice possess UV and M cones (*e.g.*, Zhao et al., 2017), and another study names them S and L cones respectively (*e.g.*, Ekesten & Gouras, 2005). Therefore, we reclassified the photoreceptor inputs in the database to match the findings of photoreceptor studies. This ensures the most accurate representation of opponent cell inputs, and aids in making comparisons between studies. Our data, and the code used to generate figures, can be found online at: https://osf.io/cm689/, DOI: https://doi.org/10.17605/OSF.IO/CM689.

## Background

Opponency can be found throughout the vertebrate visual system, which is composed of the eyes and brain. Within the eye, information typically passes unidirectionally from the outermost retinal layers to the innermost retinal layers (Fig. S1B) (Gouras, 2009). From upstream to downstream, the cell layers of the retina are as follows: photoreceptor layer, horizontal cell layer, bipolar cell layer, amacrine cell layer, and retinal ganglion cell layer. These retinal layers are conserved across vertebrate species (Hahn et al., 2023). From the retinal ganglion cell layer, information leaves the retina and moves to different brain regions based on the species. While one may think of the retina as a distinct sensory organ, separate from the brain, it is in fact an extension of the brain within the eye (Dowling, 2012). Light information also travels to non-image-forming brain regions where it is used to drive pupillary reflexes, seasonal behavior changes, and circadian rhythms (Schmidt & Kofuji, 2008). This review focuses on the image forming regions of the visual system.

Opponency requires the comparison of signals from multiple photoreceptors (Fig. S1A (Gouras, 2009). Photoreceptors can be divided into two classes: rods and cones. Rods are named for their cylindrically shaped outer segments. Rods are typically active in low ambient light conditions which enables them to contribute to night vision and detection of luminosity signals (Dowling, 2009). There are three types of cones that are commonly found in the vertebrate visual system: single, twin, and double cones (Baden et al., 2025; Walls, 1963). Cones are named for their tapered outer segments and are typically active in high ambient light conditions. Single cones have the simplest configuration of all cone types: one outer segment, one inner segment, one cell body, and one synaptic terminal. Vertebrates are reported to possess between one and five types of single cones that are sensitive to different portions of the visible spectrum (Dowling, 2012; Baden et al., 2025; Sabbah et al., 2010). We follow the cone nomenclature recommended by De Valois (1972) where photoreceptors are assigned a letter based on the portion of the visible spectrum to which they are most sensitive. These are: L for long wavelength (red) sensitive cones, M for medium wavelength (green), S for short wavelength (blue), V for violet, and UV for ultraviolet sensitive cones (however, see Baden et al. (2025) for a recent revised nomenclature). The other types of cones, twin and double cones, have two conjoined cone members (Kelber, 2025). Both members of twin cones are the same size (Walls, 1963). Twin cones may have the same photopigment, usually red/red or green/green (Burkhardt et al., 1980; Hawryshyn et al., 1988; Nagloo, Hart & Collin, 2017), or different photopigments, usually red and green (Kelber, 2025). Double cones, however, are morphologically asymmetric: the primary member is larger and the secondary member is smaller (Thoreson & Dacey, 2019; Seifert, Baden & Osorio, 2020). These members of double cones sometimes have different photopigments and are sensitive to different wavelengths of light (Thoreson & Dacey, 2019). Other times, both members are sensitive to the same wavelengths (Seifert, Baden & Osorio, 2020; Grötzner et al., 2020). In the literature there is not always a clear distinction between the two types of paired cones—oftentimes the label “double cone” is used to describe both types (Thoreson & Dacey, 2019; Kelber, 2025; Baden & Osorio, 2019; Carleton et al., 2020). We use the phrase “paired cone” when discussing both types. Paired cones are hypothesized to serve many functions, including daylight luminosity vision and motion detection.

The first step of opponent processing occurs in the photoreceptor layer. Photoreceptors signal (reduce glutamate release) when they detect light (Schwartz, 2021). The strength of the signal depends on the spectral properties of the photoreceptor pigment and the spectral properties of the light stimulus (Thoreson & Dacey, 2019). When two photoreceptors with different spectral sensitivities are illuminated with the same light stimulus, they produce different responses (Fig. S1A). The comparison of these different responses enables the visual system to determine the spectral components of the light (Daw, 2009). When these light signals are summed, they produce a luminance signal (Fig. S1Ai). When they are subtracted, *via* cone opponent mechanisms, they produce a chromatic signal (Fig. S1Aii) (Kraaij, Kamermans & Spekreijse, 1998; Niwa & Tamura, 1969; Orlov & Maksimova, 1965). Opponent cells respond to changes in wavelength with excitation or inhibition, driven by different photoreceptors. The type of response opponent cells exhibit is known as spectral opponency (Thoreson & Dacey, 2019). The neural mechanism which leads to this response is known as cone opponency (Thoreson & Dacey, 2019).

Cone opponent cells receive excitatory input from one or more photoreceptors as well as inhibitory input from one or more other photoreceptors (Thoreson & Dacey, 2019). Alternatively, they respond to the onset of light which excites one or more photoreceptors and to the offset of light which stimulates one or more other photoreceptors (Thoreson & Dacey, 2019). We use “cone mechanisms” to refer to the descriptive characteristics of a cone opponent cell. Cone opponency necessitates spectral opponency. All cone opponent cells exhibit spectrally opponent properties. However, the converse is not always true (Kraaij, Kamermans & Spekreijse, 1998). There are a few select cases where spectrally opponent cells receive excitatory input and inhibitory input from the same population of photoreceptors (but with different ratios of input). For example, one study of goldfish (*Carassius auratus*) horizontal cells found a spectrally opponent cell with a null point between 621 nm and 700 nm (Kraaij, Kamermans & Spekreijse, 1998). This cell has both excitatory and inhibitory cone inputs from L, M, and S cones, but the weight of the cone signals is varied (Kraaij, Kamermans & Spekreijse, 1998).

Spectral opponency is observed when a cell is excited by some wavelengths of light and inhibited by other wavelengths of light (Thoreson & Dacey, 2019). Alternatively, they may respond with the onset of some wavelengths of light and to the offset of other wavelengths of light (Thoreson & Dacey, 2019). We observe three types of spectrally opponent cells, based on the cell responses to monochromatic light. Each spectrally opponent cell has at least one peak, one trough, and one null point (Fig. S1C) (Twig, Levy & Perlman, 2003). The peak corresponds to the wavelength which maximally excites the cell, the trough corresponds to the wavelength which maximally inhibits the cell, and the null point is the wavelength in between where the cell does not respond with excitation or inhibition (Twig, Levy & Perlman, 2003). The number of null points determines the type of opponent cell (Fig. S1D): Biphasic cells have one null point, triphasic cells have two null points and tetraphasic cells have three null points (Twig, Levy & Perlman, 2003). When animals have multiple opponent cells, the different classes of opponent cells can be distinguished by the wavelength of the null point (Twig, Levy & Perlman, 2003). We use “spectral properties” to refer to the descriptive characteristics of a spectrally opponent cell.

Each opponent cell has a region of stimulus space to which it responds to its preferred stimulus, which is known as the receptive field (Daw, 1973; Jacobs, 1969). The preferred stimulus of each opponent cell has three properties: chromatic, spatial, and temporal. First, the cell chromatic properties have already been described above. They are either the type of cone inputs (cone opponency), or the wavelengths of light to which the cell responds (spectral opponency). Second, opponent cells differ in their spatial properties (Dowling, 2012). The simplest arrangement, which we call spatially uniform opponent cells, have the same cone inputs and respond to the same wavelengths of light throughout their entire receptive fields. Other cells, which we call spatially complex, have different cone inputs and respond to different wavelengths of light depending on the position within the receptive field. Typically, spatially complex opponent cells have a center–surround arrangement, where their receptive field is divided into a central region and a surrounding ring (Daw, 1973). This center–surround organization enhances the contrast of boundaries of objects in space, responding to large color patches or colored boundaries (Dowling, 2012; Shapley & Hawken, 2011). Other types of spatial complexity can occur, such as responding to full field illumination or an additional, enlarged surround ring (Jacobs, 1969; Bäckström, Hemilä & Reuter, 1978). Third, the temporal properties of opponent cells depend on the duration of cell responses to light, with cells often classified as transient or sustained (Zhao et al., 2017). Transient cells respond only very briefly to the onset or offset of light; whereas sustained cells respond to the entire duration when the light is on or off.

This review seeks to expand our understanding of opponent cells in three ways. We first report which taxa and visual system regions are known to use cone opponency for color processing. Next, we review the sampling effort applied in existing studies and discuss the limitations imposed by experimental design. Lastly, we describe how opponent cell types are currently classified and propose new methods to classify opponent cell types.

**Figure 1:**
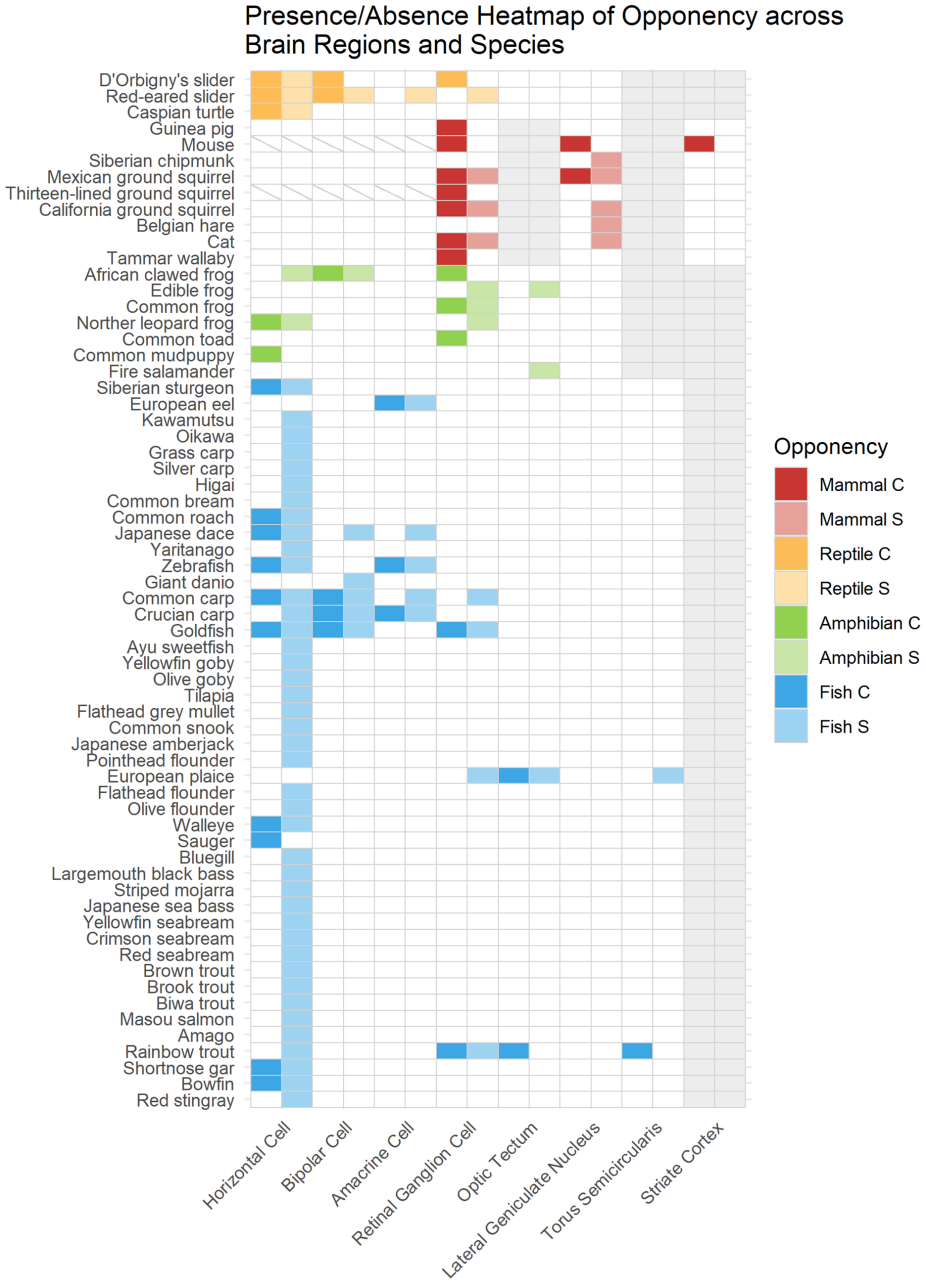
Taxonomic distribution of opponency. Opponent cell properties reported in 64 species and eight visual system layers. Colored filled cells indicate that either cone mechanisms “C” or spectral properties “S” have been reported. White cells indicate that opponency has not been reported in these species and visual system layers. Grey cells indicate that the visual system layer is not present or not involved in vision in these species. The hue of the cell indicates the major vertebrate class to which each species belongs. Cells with diagonal grey lines show regions and species where opponency has been searched for and not found.

## Taxonomic Distribution of Opponency Across Species

### Opponency in major vertebrate classes

The study of opponency has been biased towards particular vertebrate classes. In non-primate vertebrates, opponency has been studied in 64 species from four major vertebrate classes: nine mammals, three reptiles, seven amphibians, and 45 fishes. And of the fishes studied, there is a strong bias towards Japanese fishes. Further, opponency has been studied inconsistently across the various visual system regions in each major vertebrate class. The horizontal cell and retinal ganglion cell layers have been studied more than other layers (Fig. 1). Additionally, the knowledge of opponency across visual system layers within a species is sparce. Multiple visual system layers have been described in only 16 species (Fig. 1). The types of photoreceptors involved in the neural mechanisms of opponency differ based on species, but some trends within and across major vertebrate classes emerge.

To elucidate the boundaries of our current knowledge of opponent cells, we assess trends within each major vertebrate class studied. The abundance of data on fishes allows us to explore the visual system regions more thoroughly. Additionally, we describe what is known about opponency in birds, despite the lack of a complete description of any opponent cells opponency in any avian species. Then, we focus on species for which more than one visual system region was studied to examine changes in spatial and chromatic signals as they are transmitted downstream. Lastly, we examine the types of photoreceptors shown to be involved in opponency for each major vertebrate class. Together, these objectives enable us to identify where more research is needed.

#### Mammals

Apart from primates, opponency has been described in nine mammal species from five families (Fig. 1). The Sciuridae family (squirrels) has been the most studied with four species investigated. The cone mechanisms have been described in seven species, and the spectral properties have been described in five (Table S1). The retinal ganglion cell layer was the most studied in mammals. All opponent cells described in mammals exhibited biphasic spectral responses (Table S2). Opponency was not described in horizontal, bipolar, or amacrine cells in any mammalian species (Fig. 1). In fact, despite extensive search, only non-opponent horizontal cells have been found in any mammal species (Schwartz, 2021). The first opponent cells, retinal ganglion cells, can have simple or complex (*i.e.,* center–surround) receptive fields.

#### Reptiles

Despite the well-studied color driven behavior of anoles and geckos, their visual neural mechanisms remain unexplored (Maximov & Wadman, 2017); opponency has only been reported in three species of reptiles, all turtles, from two families (Fig. 1). Cone mechanisms and spectral properties were described in all three species, and only within the retina. Horizontal cells were the most studied visual system layer.

Unlike in mammals, reptile opponent cells exhibited diverse spectral responses. In the horizontal cell layer, 90% of opponent cells exhibited biphasic responses, and 10% exhibited triphasic responses (Table S2). However, the distribution changes in the retinal ganglion cell layer: 44% exhibited biphasic responses, 33% triphasic responses, and 22% exhibited tetraphasic responses (Table S2). Rocha et al. (2008) reported twelve types of opponent cells based on their spectral responses in the amacrine and retinal ganglion cell layers of the red-eared slider (*Pseudemys scripta elegans*).

Additionally, opponency has not been investigated in the image-forming regions of the reptile brain. However, we know that some reptiles use opponent mechanisms in their non-image-forming pathways. The desert night lizard (*Xantusia vigilis*) and the side-blotched lizard (*Uta stansburiana*) have small photoreceptive patches atop their heads, known as the parietal eye. This connects to the pineal gland and entrains the circadian rhythm (Solessio & Engbretson, 1993; Su et al., 2006). Opponent interactions between photopigments within the photoreceptors of the parietal eye enable the detection of the day-to-night transition (Solessio & Engbretson, 1993; Su et al., 2006).

#### Amphibians

Opponency has been described in seven amphibian species from five families. The cone mechanisms have been identified in five species, and the spectral properties in six (Table S1). The retinal ganglion cell layer has been the most extensively described region in amphibians. No tetraphasic spectral responses were reported in amphibian opponent cells (Table S2). Only one trend is of note: rod photoreceptors were involved in opponency across all amphibian species and all visual system layers that were studied (see “Taxonomic Distribution of Opponency Across Species”, ‘Photoreceptors involved in cone opponency’ for more information).

#### Fishes

Opponency has been most extensively described in fishes. Forty-five species of fishes from 23 families have had an aspect of their opponent mechanisms characterized (Fig. 1). The cone mechanisms have been described in 16 species and the spectral properties in 44 species (Table S1). The horizontal layer was the most studied across fishes (Fig. 1).

Horizontal cells have been sampled in 42 species of fish. Across all spectral responses reported in horizontal cell layers, 60% exhibited biphasic responses, 36% triphasic responses, and 3% exhibited tetraphasic responses (Table S2). In the horizontal cells of fishes, the presence of spectral opponency was used as an indicator that the animal possessed color vision (Niwa & Tamura, 1969). The lack of opponency in the horizontal cell layer has been noted in some species of fish, typically fishes that are crepuscular (*Acanthopagrus schlegelii*, *Channa argus*), nocturnal (*Heterodontus japonicus*), or inhabit deep water (*Evynnis tumifrons*, *Thunnus albacares*) (Niwa & Tamura, 1969; Kawamura et al., 1981; Tamura & Niwa, 1967). (For a review of ecological correlates of types of opponency see Niwa & Tamura, 1969). Since 1969, even more fish species have been studied at the horizontal cell layer. This provides the opportunity for future comparative analyses assessing the association between the presence/absence of opponency with different environmental and ecological factors. The overabundance of fish horizontal cells studied may be attributed to the ease of sampling such cells (Daw, 1973). Fish horizontal cells are large, and thus easier to record from than photoreceptor or bipolar cells (Daw, 1973). For more on how cell size impacts opponent cell encounter rates, see “Sampling Opponent Cells”, ‘Experimental design elements influencing opponent cell discovery’.

Bipolar cells have been less well sampled, with opponency only described in five species. The cone properties have been described in three species, and spectral properties in all five. Across all the spectral responses reported in the bipolar cell layer, 84% exhibited biphasic responses and 15% exhibited triphasic responses (Table S2).

Opponency has been described in amacrine cells of five species of fish. The cone mechanisms have been described in three species and the spectral properties in all five. Amacrine cells are the most diverse cell type in the vertebrate retina; 70 morphologically distinct types of amacrine cells have been found in fish (Wagner & Wagner, 1988). The cone opponent mechanisms reported were all spatially uniform in all three species investigated (European eel (*Anguilla anguilla*): (Byzov et al., 1998); Crucian Carp (*Carassius carassius*): (Kaneko, 1973); Zebrafish (*Danio rerio*): Torvund et al., 2017). Of the spectrally opponent amacrine cells described in five species of fish, 71% were biphasic, 14% were triphasic, and 14% were tetraphasic (Table S2).

#### Birds

Although opponent properties have not been completely described in any avian species, opponent cells have been implicated in color processing in pigeons (*Columba livia*), quail (*Coturnix japonica*), and chickens (*Gallus gallus*) (Seifert, Baden & Osorio, 2020; Maturana & Varela, 1982; Wang, Jiang & Frost, 1993). According to Wang, Jiang & Frost (1993), 20% of the cells recorded from the ventral geniculate nucleus of the pigeon were opponent, and all types were biphasic. Maturana & Varela (1982) also explored the spectral properties of opponent cells in the ventral geniculate nucleus of the quail, though the inhibitory wavelengths were unspecified.

### Species with more than one visual system layer studied

Studying opponent cells across multiple visual system layers has been an essential step to determine how the visual system processes chromatic signals. Understanding the mechanisms of opponency in downstream visual system layers can be most directly achieved by recording from each element upstream (Kaneko, 1970). This incremental approach is how the fundamentals of visual processing can be decoded (Kaneko, 1970; Manu et al., 2022). When the exact connections between cell classes have been determined, wiring diagrams or connectomes can be created (Ammermüller & Kolb, 1995; Young et al., 2021). These diagrams help elucidate the functions of opponent cells and act as hypotheses for future experiments (Thoreson & Dacey, 2019; Kraaij, Kamermans & Spekreijse, 1998; Ammermüller & Kolb, 1995).

There are many gaps in our knowledge of opponent mechanisms across visual system layers; much remains to be learned (Fig. 1). Opponent cell types have been described in multiple layers in only 15 species (three mammals, two reptiles, two amphibians, and seven fishes). And opponency has been reported in all retinal layers in only two species: the red-eared slider (*Pseudemys scripta elegans*) and the common carp (*Cyprinus carpio*) (Fig. 1). Additionally, although opponency has been studied in all retinal layers of the D’Orbigny’s slider (*Trachemys dorbigni*), it was not found in all layers (Ventura et al., 2001). It may be expected that opponent cell types which occur upstream will also be found downstream (Ammermüller & Kolb, 1995; Baden, 2021). To understand if opponent cell types have the same properties through visual system layers, we examined opponent cell properties from the fifteen species that have been studied across multiple layers of the visual system (Fig. 1). We explored the spectral cell types, photoreceptor inputs, and spatial properties.

#### Spectral cell types throughout the visual system layers

If opponent cell types have the same properties across visual system layers, we expect that cell types with the same spectral properties will be reported in each visual system layer studied in the same species. For species for which the spectral properties are known, there could be between one and three spectral cell types (biphasic, triphasic, and/or tetraphasic) in each layer. The spectral cell types have been reported in multiple visual system layers for 11 species (two mammals, two amphibians, six fishes, and one reptile) (Table S3). In four species, mammals and amphibians, only biphasic response types were reported, and were consistent across all layers. In fishes and the reptile, the types of opponent cells reported change across visual system layers. There are cases where opponent cell types from the horizontal cell layer are lost downstream, such as in the zebrafish (*Danio rerio*) and crucian carp (*Carassius carassius*) (Table S3). And there are cases where opponent cell types are added downstream, like in the rainbow trout (*Oncorhynchus mykiss*) and red-eared slider (*Pseudemys scripta elegans*) (Table S3). Altogether, this illustrates that the spectral responses of opponent cells vary depending on the visual system layer.

#### Photoreceptors involved in opponency throughout the visual system layers

If cone opponent cells have the same properties across visual system layers, we would expect the same photoreceptors to be involved in opponency in every layer. Cone opponent mechanisms are known in multiple layers of the visual system for nine species (Table S4). Of these nine species, six showed the same photoreceptors contributing across all layers studied. In three species, goldfish (*Carassius auratus*), mouse (*Mus musculus*), and red-eared slider (*Pseudemys scripta elegans*), the photoreceptor contributions varied by layer. However, this may be an experimental artifact; the contribution of rods and ultraviolet sensitive photoreceptors was investigated in some layers but not in others (*e.g.*, Kraaij, Kamermans & Spekreijse, 1998). Alternatively, this may reflect different pathways for different photoreceptors. For example, Baden (2021) proposes UV photoreceptor input does not contribute to opponency until after it leaves the retina, thereby avoiding spectral filtering by early opponent mechanisms. Similarly, in rodents there are different pathways for different cone types before reaching opponent cells. Cone-type selective bipolar cells which feed into ganglion cells only receive input from M or UV cones (Ekesten & Gouras, 2005; Breuninger et al., 2011; Li & De Vries, 2006). While we currently do not have sufficient evidence to determine if opponent cell types change between layers, future investigation into UV cones’ and rods’ input into opponent mechanisms may provide clarity.

#### Spatial properties throughout the visual system layers

Lastly, we explored how the spatial properties of opponent cells change throughout the visual system layers. Opponent cell spatial complexity is expected to increase as information passes through the visual system (Daw, 1973). For species other than mammals, the first opponent cells are found in the horizontal cell layer (Fig. 1). In this visual system layer, all opponent cells are spatially uniform (Kato, 1979). All other visual system layers in the visual system pathway can have spatially uniform or complex cells. In mammals, opponency does not appear until the retinal ganglion cell layer and may already be spatially complex.

Spatial properties of opponent cells have been investigated in multiple visual system layers for 12 species (Table S4). Our observations mirror those reported in Daw (1973): horizontal cells are always spatially uniform (Table S4), all other visual system layers investigated expressed spatially simple and/or spatially complex arrangements across species. If a species exhibits both spatially uniform and spatially complex opponent cells, the spatially complex cells never appear before spatially uniform cells. They either arose concurrently or the spatially uniform cells appeared first. In seven species, the types of opponent cells varied by layer and in five species they were invariant (Table S4). However, there are many visual system layers yet unstudied. It is very possible that more specific trends will be revealed upon further investigation of the layers which remain to be explored. Regardless, it seems that the spatial properties of opponent cells are not consistent across visual system layers.

#### Functions in different layers

Ultimately, opponent cell types do not seem to persist through the visual system layers in all species studied so far. Opponent cells may serve different functions throughout the visual system. For example, it has been proposed that the outer retina, where horizontal cells reside, functions to establish the color channels, while the inner retina and other visual system layers are dedicated to spatiotemporal computations (Baden, 2021). This idea is supported by our finding that spatially complex cells are more often found in downstream layers (Table S4). Likewise, it has been suggested that opponent horizontal cells primarily function as filters which narrow the spectral sensitivity of the chromatic channels (Connaughton & Nelson, 2010). This spectral filtering may function similarly to oil droplets in birds and reptiles which have been shown to enhance color discrimination (Vorobyev, 2003). Further research should be undertaken to determine opponent properties across visual system layers more completely for these species and to increase the species pool for which multiple layers have been studied.

### Photoreceptors involved in cone opponency

According to the available data (Teed, Hartzler & Fernández-Juricic, 2024), all photoreceptors in the outer retina, with the exception of paired cones with the same photopigment in both members, are involved in opponency (Fig. S3). The inputs of double cones and rods can be disambiguated from single cone inputs, either by their spectral sensitivities or by different temporal properties (Djamgoz & Ruddock, 1979).

The role single cones play in vision is well established (Dowling, 2012). Among other functions, such as high-resolution vision, single cones are also the primary drivers of color vision. For cones to be involved in color vision, it is expected that they will contribute to opponent mechanisms; therefore, we expect each type of single cone an animal has will contribute to its opponent mechanisms (Baden & Osorio, 2019). To investigate this, we first identified the photoreceptors in all species for which we know the cone opponent mechanisms. Next, we compared the list of cones involved in opponency to the total list of cones an animal has, looking for any discrepancies. For 22 out of 30 species, all single cones are reported to be involved in opponent mechanisms. However, in eight species, animals have more single cone types than have been reported to contribute to their opponent mechanisms. The “missing” cones are always the shortest cone an animal possesses: either UV, S, or both. There are a few reasons why the UV and S cone input may not be reported. Firstly, as mentioned previously (“Taxonomic Distribution of Opponency Across Species”, ‘Photoreceptors involved in opponency throughout the visual system layers’), UV input may only contribute to opponency in further downstream layers to avoid filtering, and its presence would not be detected if downstream cells are not sampled. Secondly, it is possible that the shortest single cone serves an achromatic function and does not contribute to color processing. Thirdly, many experimental designs did not account for UV photoreceptors in their designs. Many studies used monochromators which produced light between 400 and 700 nm, which exclude UV photoreceptors. Lastly, it is possible that the opponent cells which receive input from UV photoreceptors are smaller and harder to sample, like in the torus semicircularis of the rainbow trout (*Oncorhynchus mykiss*) (Coughlin & Hawryshyn, 1994). One species, the European plaice (*Pleuronectes platessa*), was reported to have three single cones in the opponent literature, but only two single cones in photoreceptor literature (Hammond, 1968; Loew & Lythgoe, 1978).

Paired cones have been suggested to fulfil multiple roles in vision, including: detecting polarized light, motion detection, daytime luminance vision, and color vision (Seifert, Baden & Osorio, 2020; Ebrey & Koutalos, 2001; Günther et al., 2018; Lind, Chavez & Kelber, 2014; Osorio & Vorobyev, 2005). We observed that paired cones which have different photopigments in their two members have contributed to the cone mechanisms of opponency in fishes and amphibians, and paired cones with the same photopigments between the members cones have not contributed at all (see associated dataset at https://osf.io/cm689/). In fishes and amphibians, both members of the double cone contribute to the same opponent mechanisms; sometimes the members are opponent to each other and other times they are opponent to a different photoreceptor. While paired cones are not uncommon across vertebrate species, their reported involvement in opponency is infrequent and merits further investigation. Paired cones have not been reported in the photoreceptor literature for any mammalian species in which opponency was studied; even including the tammar wallaby (*Notamacropus eugenii*) which, as a marsupial, was thought to possess twin cones (Arrese et al., 2002). All reptiles studied with a confirmed paired cone possess the same photopigment in both member (Teed, Hartzler & Fernández-Juricic, 2024). One study in the red-eared slider (*Pseudemys scripta elegans*) reported double cone contributions, but as the photopigments described in the opponent literature do not match photoreceptor literature we excluded it (Fuortes & Simon, 1974) from Fig. S3.

Rods are generally involved in achromatic vision and used in low light conditions (Fein & Szuts, 1982). However, Tikidji-Hamburyan et al. (2017) showed that rods can operate in daylight conditions and contribute to image-forming vision in high-light levels. We observed rod contributions to opponent mechanisms in three of the four major vertebrate classes (Fig. S3). First, in all amphibian species studied (Fig. 1), every opponent cell which was reported included input from rods. All anurans studied for opponency have two rods—a red rod and green rod—and between one and three single cones (Ala-Laurila et al., 2002; Bäckström & Reuter, 1975; Koskelainen, Hemilä & Donner, 1994; Liebman & Entine, 1968; Reichenbach & Fuchs, 1983; Röhlich & Szél, 2000; Wilhelm & Gábriel, 1999). At photopic levels, the green rod contributed to opponent cells with cones, always resulting in opponency between a single rod and a single cone. As light levels decrease, the red rod response replaced the cone response, creating rod-rod opponent circuits which enable color vision in scotopic conditions (Bäckström & Reuter, 1975; Yovanovich et al., 2017). In mammals and fishes, rods contribute to opponency, though the contribution is reported infrequently. In mammals, rods contribute to opponent mechanisms in mice (*Mus musculus*) and Mexican ground squirrels (*Ictidomys mexicanus*) (Joesch & Meister, 2016; Tong, 1977). While opponent mechanisms are not known for any aquatic mammal species, behavioral experiments in the spotted seal (*Phoca largha*), harbor seal (*Phoca vitulina*), and African fur seal (*Arctocephalus pusillus*) imply that rods are involved in photopic color vision (Oppermann, Schramme & Neumeyer, 2016; Wartzok & McCormick, 1978). In fishes, rods contribute to opponent mechanisms throughout the visual system (Govardovskii et al., 1991; Joselevitch & Kamermans, 2007). Lastly, reptiles have never been reported to have rod contributions to opponent channels.

## Sampling Opponent Cells

As we have seen, there are many species and visual system layers for which opponency remains unknown, and there are many open avenues for opponent cell discovery. Future studies undertaking opponent cell discovery should account for appropriate sampling and experimental conditions in their design. Here, we have compiled the sample sizes and other metrics from existing studies so that future experimenters may determine the appropriate sample size for their specific species and visual system region. The calculated sample size may depend on how common opponent cells are and on how many types are expected to be found. Additionally, experimental conditions should be considered in future studies, such as the physiological state of the retina or ambient light conditions, which influence opponent cell discovery (see “Sampling Opponent Cells”,‘Experimental design elements influencing opponent cell discovery’). To address these needs, we have compiled the sampling metrics and experimental conditions which influence opponent cell discovery.

### Sampling metrics extracted

**Figure 2:**
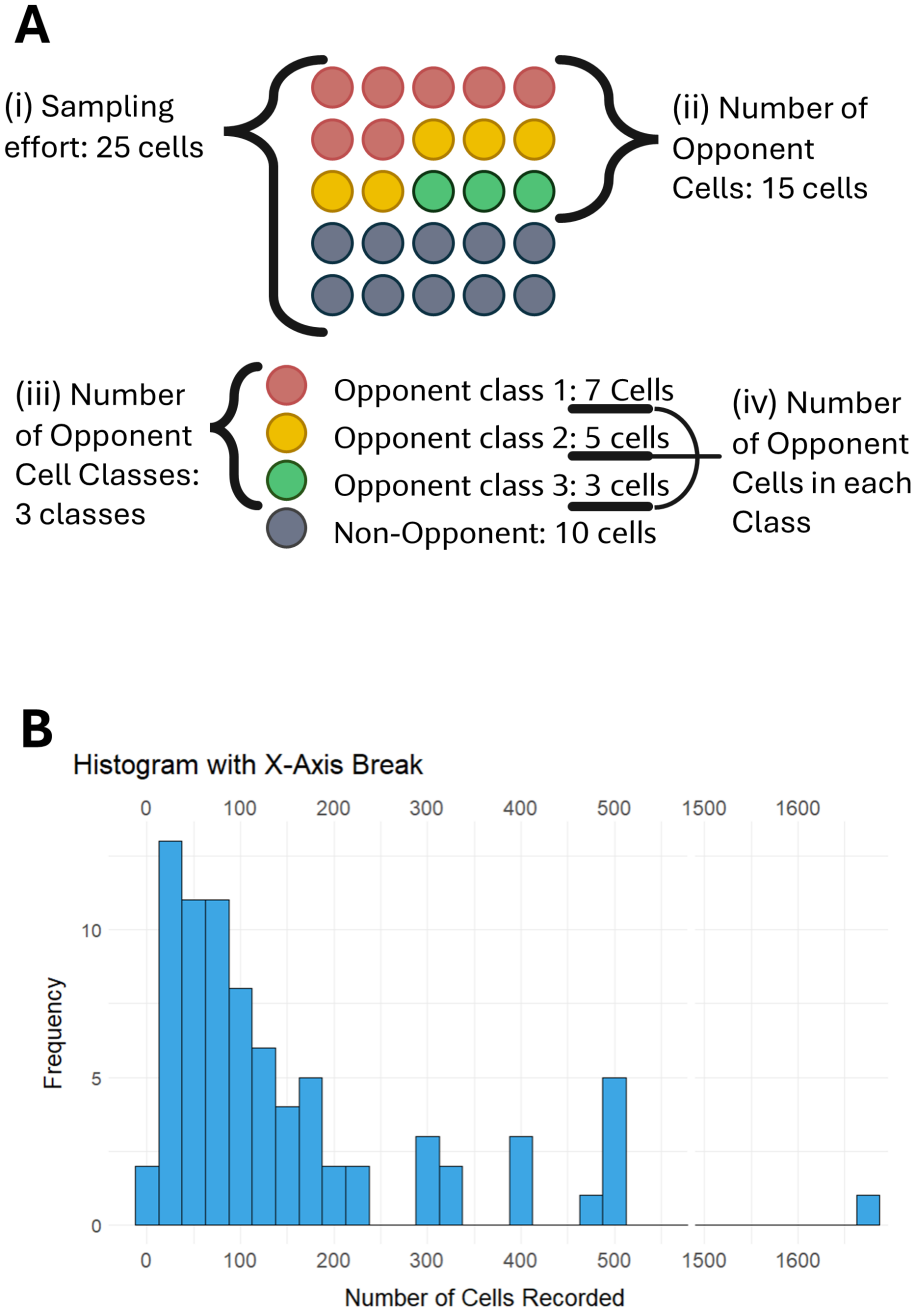
Sampling opponent cells. (A) Sampling metrics extracted. Each circle represents one opponent cell sampled. Sampling effort (Ai) refers to the total sample of cells recorded from. In this example, it is 25 cells. The number of opponent cells found (Aii) encompasses cells that are opponent. The number of opponent cell classes (Aiii) refers to the different types of opponent cells. The number of cells in each class (Aiv) sums to the total number of opponent cells (Aii). (B) Histogram of the number of cells recorded across studies of opponent mechanisms. An *x*-axis break occurs between 500 and 1,500 cells (Xu et al., 2021).

We evaluated the extent of sampling in each study which reported cone opponent mechanisms. We extracted four sampling metrics from each study:

(1) Sampling effort: the total number of cells sampled, including both opponent and non-opponent cells (Fig. 2Ai).
(2) Number of opponent cells: the number of cells sampled which exhibited opponent properties (Fig. 2Aii)
(3) Number of opponent cell classes: how many types of opponent cells are found (Fig. 2Aiii).
(4) Number of opponent cells in each cell class (Fig. 2Aiv) (Xu et al., 2021)

There may be more than one value reported for each study if the study sampled opponent cells from multiple species or multiple visual system layers.

Unfortunately, the sampling metrics were not reported by every study. Only 50% of studies reported all four sampling metrics. The number of cells sampled (1) was reported by 58% of studies, and the values range from nine cells (Ventura et al., 1999) to 1,679 cells (Yin et al., 2009) (Fig. 2B). Most commonly, studies reported a sampling effort between 25 and 50 cells (Fig. 2B). Sixty-three percent of studies reported the number of opponent cells (2) encountered, which ranged between one (Ventura et al., 1999; Fain, 1975) and 274 (Michael, 1973). The number of opponent cell classes (three) was part of the inclusion criteria for the dataset and was therefore reported by all studies. Thirty-two percent of studies only reported encountering one opponent cell class, though this may be because the study was only investigating one specific opponent cell type (*e.g.*, Djamgoz & Greenstreet, 1996). The most opponent cell classes encountered in one visual system layers and one species within a single study was 12 (Coughlin & Hawryshyn, 1994). Sixty-eight percent of studies reported the number of opponent cells in each class. Of these studies, 37% reported only one cell representing an opponent cell class. The highest number of opponent cells in a single class was 60 cells (Jacobs & Tootell, 1980).

### Opponent cell encounters and abundance

In order to determine how common opponent cells are throughout the visual system, we calculated the percentage of opponent cells encountered in each visual system layer across each species. We were able to calculate this percentage for all studies that reported the sampling effort and number of opponent cells (52% of studies). We compiled the average percentage of encounters across major vertebrate classes and visual system layers.

Across all studies, we observed that the percentage of encounters was lower in amphibians (means ± SD: 24.4 ± 26.5%) and mammals (26.5 ± 21.7%) than in reptiles (44.9 ± 22.8%) or fishes (47.2% ± .8%). Overall, the percentage of encounters ranged from 0.93% of cells exhibiting opponent properties in the mouse (*Mus musculus*) striate cortex (Ekesten & Gouras, 2008), to 93% in the retinal ganglion cell layer of goldfish (*Carassius auratus*) (Adams & Afanador, 1971). The five studies which reported the highest rates of opponent cell encounters all used teleost fishes (goldfish (*C. auratus*): (Adams & Afanador, 1971); crucian carp (*Carassius carassius*): (Kaneko, 1973; Kato, 1979); rainbow trout (*Oncorhynchus mykiss*): (Coughlin & Hawryshyn, 1994); common carp (*Cyprinus carpio*): (Mitarai, Asano & Miyake, 1974). The five studies with the lowest rate of opponent cell encounters include the non-Sciuridae mammals (mouse (*M. musculus*) (Ekesten & Gouras, 2008); guinea pig (*Cavia porcellus*): (Yin et al., 2009); tammar wallaby (*Notamacropus eugenii*): (Hemmi, Maddess & Mark, 2000) and two amphibians (northern leopard frog (*Lithobates pipiens*): (Ogden, Mascetti & Pierantoni, 1985); common mudpuppy (*Necturus maculosus*): (Fain, 1975). These findings demonstrate biological variation in the encounter rates of opponent cells.

One might expect that the percentage of opponent cells encountered corresponds to the relative abundance of opponent cells in each species and visual system layer. If this were the case, the percentage of encounters could be used to calculate the optimal sampling effort, given a desired number of opponent cells. However, we did not find any studies estimating the relative abundance of cone opponent cells to confirm this. Moreover, multiple studies within the same visual system layer and species report vastly different rates of encounters. For example, in the horizontal cell layer of the common carp (*Cyprinus carpio*), Mitarai, Asano & Miyake (1974) reported that 28% of cells encountered were opponent, whereas Kato (1979) reported 80%. Likewise, in the retinal ganglion cell layer of the Mexican ground squirrel (*Ictidomys mexicanus*), the reported rate of encounter was 24% in one study (Michael, 1968), and 52% in another (Tong, 1977). Ultimately, we cannot rule out that differences in opponent cell encounters are not a product of different experimental goals, designs, and equipment.

Knowing the relative abundance of opponent cells could inform future experimental design and potentially tell us something innate about animal visual systems. While relative abundance is not currently known for any species or visual system layer, the percentage of opponent cells encountered may be used as a starting point, if the experimenter carefully considers the impact of any difference in experimental goals, designs, and equipment between their study and existing work. Correspondingly, relative abundance can also be used to make cross-species and cross-region comparisons about visual system strategies. While the relative abundance is currently unknown for any species or visual system layer, it may be worth investing in estimating the relative abundance in species and visual system layers for which we already know the opponent cell complement.

### Experimental design elements influencing opponent cell discovery

There are a variety of experimental and physiological conditions which can influence opponent cell discovery. These conditions may either change the rate of opponent cell encounters or may change the types of opponent cells which are present (Daw, 1973). Additionally, establishing the correct parameters ensures viability of the retinal tissue (Niemeyer, 1981). It is up to the experimenter to ensure that the lighting, electrode types, and environmental conditions are carefully selected.

Experimental lighting has been shown to affect opponent cell discovery. Obviously, the light levels during experimentation impacts the types of opponent cells found, as not all photoreceptor types are active at the same ambient light levels (Dowling, 2012; Fein & Szuts, 1982). Changing the intensity of the light stimulus also changes the spectral response of the cells and shifts the null point (Twig, Levy & Perlman, 2003). If the position of the null point is the metric used to classify the opponent cell types, then the null point shifting position at different light levels may make it appear as though there are more opponent cell types than truly exist (Twig, Levy & Perlman, 2003). Additionally, the lighting conditions during an animal’s development impacts the development of photoreceptor ratios (Guinea pig: Hu et al., 2011). The types and ratios of opponent cells have been shown to vary when animals were reared in different light environments (Cichlid fish: Kröger, Braun & Wagner, 2001; Ground Squirrel: McCourt & Jacobs, 1983).

In single electrode recording studies, the relative size of opponent and non-opponent cells can influence the relative encounter rate. Smaller cells have a larger resistance and may be underrepresented in the sample (Towe & Harding, 1970; Stone, 1973). Coughlin & Hawryshyn (1994) observed that UV sensitive opponent cells in the torus semicircularis of the rainbow trout (*Oncorhynchus mykiss*) were larger than other cells and therefore may be overrepresented. Similarly, non-opponent bipolar cells are larger than opponent bipolar cells in fish and turtle retina (Yazulla, 1976). This means that if opponent cells are smaller than non-opponent cells, it could lead to a lower frequency of encounters. The direction the electrode approaches from can also impact opponent cell encounter rate in the horizontal cell layers; the non-opponent cells are localized to the most exterior sublaminae (Kato, 1979; Mitarai, Asano & Miyake, 1974; Toyoda, Kujiraoka & Fujimoto, 1982).

The physiological state of the animal also impacts the classes and ratios of opponent cells present. In the ground squirrel (*Ictidomys mexicanus* and *Ictidomys tridecemlineatus*), the animal’s blood pressure is correlated to changes both the spatial and chromatic properties of opponent cells (Gur & Purple, 1978). There are more types of opponent cells found when the animal’s blood pressure is lower. Other physiological parameters, such as PCO_2_, respiratory rate, and heart rate did not show strong correlations with opponent cell encounter rates (Gur & Purple, 1978; Gur & Purple, 1976).

Normally, the chemical microenvironment of the retina is maintained by the retinal pigmented epithelium (Sparrow, Hicks & Hamel, 2010). However, in experiments with an isolated retina, it is up to the experimenter to provide oxygen, remove metabolic waste, maintain a healthy pH, and ensure the availability of necessary ions for the neurons to function. Selecting these parameters is no mundane feat and can have either direct or indirect consequences on opponent cells (Niemeyer, 1981). When there is a reduction in oxygen concentration at the retina, retinal tissues can quickly become damaged (Niemeyer, 1981). It has been shown that horizontal cell and amacrine cell response thresholds are sensitive to shifts in CO_2_ concentration, anoxia, and the presence of NH_3_ (Negishi & Svaetichin, 1966b). Different species may be more tolerant of a range of chemical conditions, such as cyprinid teleosts, turtles, and amphibians, which enables them to recover from periods of hypoxia (Amaral-Silva & Santin, 2023; Cerra et al., 2023; Krivoruchko & Storey, 2010). The concentration of oxygen in the retina impacts the types of opponent cells which are present. In goldfish (*Carassius auratus*), increasing the concentration of oxygen in the perfusion media had the following effects: the sensitivity of the “red” chromatic system was increased, and the peripheral portion of the receptive field disappeared, thereby reducing spatial complexity (Daw, 1973; Abramov & Levine, 1972; Spekreijse, Wagner & Wolbarsht, 1972). Maintaining the appropriate chemical environment is essential for opponent cell discovery.

Experimental temperature has also been shown to impact the sensitivity of the visual system (Daw, 1973; Negishi & Svaetichin, 1966a), therefore the experimenter should select their temperature conditions with care. Many experiments are conducted below body temperature, as this prolongs tissue viability (Niemeyer, 1981). Additionally, the retina of some species is able to recover from short term refrigeration after extraction (European eel (*Anguilla anguilla*): (Byzov et al., 1998; Damjanović et al., 2005); red-eared slider (*Pseudemys scripta elegans*): (Ammermüller & Kolb, 1995; Ventura et al., 1999); Japanese Dace (*Tribolodon hakonensis*): Hashimoto & Inokuchi, 1981), which enables an animal’s second retina to be preserved while the first is being sampled. Species which can tolerate long-term anoxic conditions are also more suited to withstand freezing temperatures (Storey & Storey, 1988). Therefore, cyprinid teleosts, turtles, and amphibians are also especially tolerant to changes in temperature (Amaral-Silva & Santin, 2023; Cerra et al., 2023; Krivoruchko & Storey, 2010). However, tolerance to a variety of temperatures does not mean that opponent cell sampling will not be impacted by shifts in temperature. The opponent cells of the goldfish (*Carassius auratus*) retina, a cypranid teleost, are particularly sensitive to temperature changes. The ratio of opponent cells to non-opponent cells is higher at 17 °C than at 12 °C (Spekreijse, Wagner & Wolbarsht, 1972). This shift is linked to the activity levels of the “green” sensitivity system (Spekreijse, Wagner & Wolbarsht, 1972). The impacts of this shift have even been observed in psychophysical experiments: goldfish are less sensitive to greens at 15 °C than 25 °C (Thorpe, 1971; Thorpe, 1973). Even though a retina may be viable for recording across a wide range of temperatures, the ratios of opponent cells found may not be consistent.

Ultimately, different experimental conditions have been shown to influence the sampling of opponent cells. Inconsistent light levels during experiments, lower blood pressure levels, and increase in oxygen concentration have been shown to increase the number of opponent cell classes. Larger cell size and higher temperature may increase the ratio of opponent to non-opponent cells. Lastly, the spatial complexity of the opponent cells is susceptible to change with changes in oxygen concentration and blood pressure. The retina, including opponent cells, is particularly sensitive to deviations from normal homeostatic parameters.

### A call for more transparent reporting

Our ability to draw strong conclusions about the impact of sampling on opponent cell discovery is significantly limited. Firstly, any sampling trends we find are based on an incomplete dataset, as only 50% of studies report all their sampling metrics (Fig. 2B). It is possible that if we had access to the sampling metrics for every study we would find different trends for opponent cell ratios. Additionally, gaps in our knowledge of encounter rates occur because sampling metrics have never been reported in some major vertebrate classes and visual system layers. We currently do not have enough information to suggest sample sizes for future experiments based on relative abundance nor on the relationship between sampling size and number of cell classes encountered.

Going forward, we propose changes to the reporting of experimental details to enhance reproducibility and clarify future experimental design. First, we suggest that all studies report the four aforementioned sampling metrics: sampling effort, number of opponent cells, number of opponent cell classes, number of opponent cells in each cell class (Fig. 2A). This will also provide more estimates of opponent cell encounters, which will aid in the design of future studies. Reproducibility, data interpretation, and comparisons between studies can also be improved by reporting other aspects of the methods, such as the physiological conditions of the experiment, as these aspects impact opponent cell discovery.

## Opponent Cell Classifications

### Existing opponent cell identifiers

When extracting opponent cell mechanisms and spectral sensitivities from the literature, we observed a broad swath of labels used to classify opponent cells. These labels were frequently descriptive, providing varying degrees of detail regarding the cells’ opponent properties. However, it is also common for cells to be identified by labels which do not correspond to their opponent properties.

Most often, opponent cell labels that carry information about the cell opponent properties incorporate a representation of color in the naming (red/green & green/blue (Fuortes & Simon, 1974), RG & YRB (Kato, 1979), R+G-, R-G+B-, & R-G+ (Shimbo et al., 2000), +B-G & -B+G (Wakakuwa et al., 1987)). This is due, in part, to changing naming conventions of photoreceptor types (Daw, 1973), where photoreceptors were previously named based on the color appearance of the wavelength they were most sensitive to. Sometimes, the modern correlate of photoreceptor inputs have been used instead (Ventura et al., 2001: L/M, S/LM, UVSM+L- center and UVSM-L+ periphery, UV- SML+, *etc*). Additionally, opponent cell labels often convey the polarity associated with each photoreceptor input or wavelength of light. This polarity may be conveyed using the +, -, and ± symbols, or by the order in which photoreceptor inputs are arranged (*e.g.*, everything before the/is excitatory and after is inhibitory) (*e.g.*, Ventura et al., 2001; Yazulla, 1976). Alternatively, many studies assign all opponent cells the labels “C1-C4” (*e.g.*, Tamura & Niwa, 1967). C refers to C-type, or color-type, cells. This is contrasted with L-type (luminance) cells. Lastly, some cells are labeled based on their spectral properties (Biphasic Horizontal Cell & Triphasic Horizontal Cell) (*e.g.*, Kawamura et al., 1981). These identifiers give some description of how we expect the opponent cell to respond to light.

In other cases, the identifiers used are less descriptive of the cell’s opponent function or properties. First, some cell identifiers are based on the cell morphology, rather than the cell responses (Rocha et al., 2008). Likewise, some cells are identified primarily by their neuroethological functional properties (Maturana & Varela, 1982; Scheibner, Hunold & Bezaut, 1975). Other studies may first identify the cells based on experimental elements. Ventura et al. (2001) and Ammermüller & Kolb (1995) use a labeling system which includes the animal ID, the eye recorded from (left or right), and cell type (*e.g.*, ganglion cell, amacrine cell, bipolar cell). These identifiers may be used in conjunction with functional identifiers.

In summary, identifiers assigned to opponent cells are study-specific and complicate broad comparisons across visual system layers and species. The examples above are a mere sample of the diversity of opponent cell identifiers. While some interspecific comparisons are possible when studies use the same naming conventions, there is no straightforward way to compare Class III opponent cells to red/green cells. Changes in photoreceptor naming conventions have also introduced ambiguity into the comparison between R/G and L/M types. Indeed, photoreceptor naming conventions may change yet again (Baden et al., 2025). Yet, there is a need for a classification system which can be broadly applied to existing studies. Therefore, we seek to provide a uniform framework for classifying opponent cells based on their photoreceptor inputs and spectral responses.

### Three simple classifications

Here, we present three classification systems which can be used to assign descriptive labels to opponent cells, regardless of species, based on their photoreceptor inputs. Each label conveys two types of information about the photoreceptor inputs: their relative sensitivity in terms of wavelength, and their relative polarities. Explicitly, cone identities are L for long wavelength sensitive single cones, M for medium wavelength, S for short wavelength, UV for ultraviolet sensitive single cones, R for Rods, and DCp and DCs for the primary and secondary members of the double cone, respectively. Further, the polarity indicates how the photoreceptors drive the opponent cell response. Cones with a positive polarity (+) excite the cell, depolarize the cell, and/or drive the cell on-response. Cones with a negative polarity (–) inhibit the cell, hyperpolarize it, and/or drive the cell off-response. Lastly, if a cone contributes to both excitatory and inhibitory responses in the same cell the polarity is represented with the plus-minus symbol (±). The ± response is found in spatially complex opponent cells, and is described in more detail in “Opponent Cell Classifications”, ‘Spectral distinctions: enhancing clarity with null points’. All three classification methods use the same types of information because they build off each other. However, ultimately, they will reveal different patterns. The explanations we provide here apply to all spatially simple cells. An explanation on how to classify spatially complex cells can be found in the supplementary materials.

#### Historic classification

**Figure 3:**
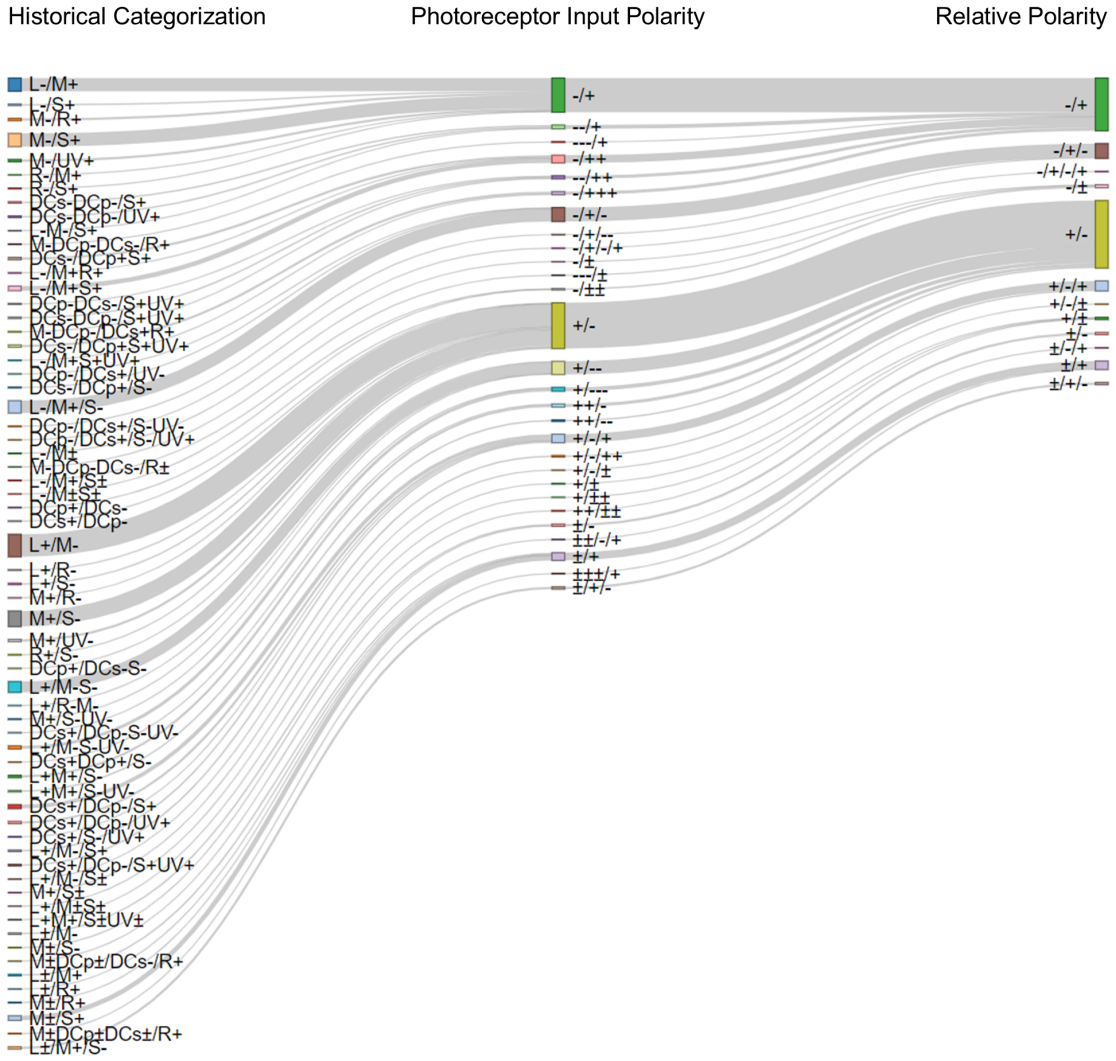
Classifying cone opponent cells with three methods. Sankey plot illustrating how the same opponent cell can be described using the three classification methods. The three columns show opponent cells classified according to the three classification methods. In the first column, each type of cone opponent cell is labeled in text form following the Historical Categorization. The middle column shows the Photoreceptor Input Polarity classification for the same cells. The last column shows the relative polarity category. Grey lines indicate how the same cell type classified across multiple methods and the height of the block indicates the number of cells classified this way.

This classification method approximates the method first described by Daw (1973); we define it as the historic method and advocate a modified version for general use. First, the label assigned to each cone opponent cell requires knowledge of the photoreceptor identity (*i.e.,* cone, rod, double cone) and photoreceptor peak sensitivity. The photoreceptors are arranged in descending order of peak sensitivity. For example, an opponent bipolar cell which contacts an L cone with a peak sensitivity of 611 nm and a rod with a peak sensitivity of 445 nm, as in *Xenopus laevis*, will have the photoreceptor inputs arranged LR (Teed, Hartzler & Fernández-Juricic, 2024; Wilhelm & Gábriel, 1999). Next, the polarity of the input is associated with each photoreceptor. Continuing the *X. laevis* example, the opponent bipolar cell on-response is driven by the L-cones, and the off-response is driven by the rods. Therefore, this cell’s inputs are L+ and R-. Lastly, slash “/” is inserted between cone inputs, like a null point, to denote a change in polarity: L+/R-. This classification method is the most detailed and can be used to identify how specific cone types contribute to opponent cells. For example, rods contribute to excitation in 12 opponent cells and inhibition in nine cells (Fig. 3).

We classified the opponent cells from the Teed, Hartzler & Fernández-Juricic (2024) database using the historic classification. All opponent cells for which photoreceptor inputs are known were sorted into 64 cell types (Fig. 3). Forty-one of these cell types were only reported once. The most common cell type was L+/M-, with 29 opponent cells.

#### Photoreceptor input polarity classification

This classification method establishes the number of photoreceptor inputs along with their polarity but is agnostic to specific photoreceptor identity. This classification method starts identically to the historic classification method. Photoreceptor inputs are arranged in order, from longest wavelength to shortest (L, M, S, *etc*.), and polarities are assigned to each photoreceptor. Next, the cone identities are removed. This means that the L+/R- cell above will be renamed to +/- in the cone input polarity method. This method is particularly informative in two cases. First, this method distinguishes opponent cells that have more than one photoreceptor input into each phase. For example, in the common toad (*Bufo bufo*), the retinal ganglion cell which is classified as M-DCp-/DCs+R+ under the historical method is classified as –/++ under the cone input polarity method. This may be useful to disambiguate between biphasic cells with a similar response profile (*e.g.*, ++/- *vs* +/-). Similarly, this method retains information about the number of photoreceptor inputs, without introducing noise based on the specific photoreceptor types. Therefore, this method is particularly useful for drawing interspecific comparisons about opponent cells when the species being compared do not possess the same photoreceptor classes.

When we classified the opponent cells from the Teed, Hartzler & Fernández-Juricic (2024) database using the photoreceptor input polarity classification, all 237 cells from 44 species were sorted into 27 cell types (Fig. 3). The most prevalent cell type was +/- cells, which had 59 cells. Twelve cell types were only represented by a single cell.

#### Relative polarity classification

Our final classification method is agnostic to the number of photoreceptors and to photoreceptor identity. Cone inputs are arranged, and polarities are assigned using the conventions outlined for the previous two classification methods, but adjacent polarities are simplified. ++, –, and ± ± reduce to +, -, and ± respectively. As an example, the aforementioned common toad (*Bufo bufo*) retinal ganglion cell, which is classified as M-DCp-/DCs+R+ under the historical method, and as –/++ under the cone input polarity method, will be -/+ under the relative polarity method. This method describes the relative position of the peaks and troughs in the electromagnetic spectrum for opponent cells, and most directly mirrors the cell’s functional response to light.

When we classified the opponent cells from the Teed, Hartzler & Fernández-Juricic (2024) database using the relative polarity classification, all 237 cells from 44 species were sorted into 12 cell types (Fig. 3). Eighty-seven opponent cells fall into the most common category: +/-. Seventy cells fall into the second most common category: -/+. Only three categories represent a single cell.

This method may also be used to classify opponent cells for which the photoreceptor inputs are unknown, but spectral responses have been reported. Spectral responses may be biphasic, triphasic, or tetraphasic (Fig. S1D). This method ascribes polarities to each phase (+, -, or ± in limited cases). Therefore, an opponent cell which depolarizes to red light and hyperpolarizes to green light would be represented as +/-, even though the photoreceptors that drive this response are unknown.

#### Comparing methods

When comparing the three types of classifications within an individual species, an interesting story may emerge. According to Govardovskii et al. (1991), Siberian sturgeon (*Acipenser baeri*) have five types of opponent cells which express five spectral responses based on the position of the null point, but one type of opponent cell is divided into two subclasses based on different photoreceptor inputs (Table 1). When these cells are classified according to the historical classification method, the Siberian sturgeon (*A. baeri*), has six types of opponent horizontal cells, better reflecting the diversity of photoreceptor inputs. This method retains the most complexity. When the complexity is reduced in the photoreceptor input polarity method, there are fewer cell types (four cell types), but interestingly: the longest wavelength input is always excitatory (Table 1). Finally, the relative polarity method shows that five out of the six types of opponent cells are expected to be biphasic. Each of these trends can be seen within the historical classification; but when many species and visual system layers are being compared at scale, the photoreceptor input polarity and relative polarity methods facilitate parsing these trends.

**Table 1:** Classifying opponent cells with three methods. We classified the opponent horizontal cells in the Siberian sturgeon (*Acipenser baeri*) reported by Govardovskii et al. (1991), Wartzok & McCormick (1978) according to the three methods. Horizontal Cell Type refers to the cell classification in the original paper, with subscripts 1 and 2 added to disambiguate the subtypes of R/G cells. The null point(s) refer to the wavelength(s) between the cell’s excitatory and inhibitory responses, where it does not respond to light. The remaining three columns are how these cells would be classified according to the three methods we propose.

Horizontal cell type	Null point	Historical classification	Photoreceptor input polarity	Relative polarity
R/G_1_	600 nm	L+/M-	+/-	+/-
R/B	560–570 nm	L+/S-	+/-	+/-
G/B	540 nm	M+/S-	+/-	+/-
R/G_2_	600 nm	L+/R-M-	+/–	+/-
RG/B	520 nm	L+M+/S-	++/-	+/-
G/BR	620 nm, 640 nm	L-/M+/S-	+/-/+	+/-/+

We suggest that the historical classification method be used for reporting opponent cell types, and all three methods be used for analysis. To ensure that the number of cell types is persevered, despite the loss in complexity through the methods, the null points can be reported in conjunction with the classification. Next, we will describe how the null point can be used to enhance clarity when reporting opponent cell types.

### Spectral distinctions: enhancing clarity with null points

A limitation of these classification methods, especially with the photoreceptor input polarity and relative polarity methods, is that different types of opponent cells within the same species may now be represented using the same label. Even using the most detailed method, the historical classification method, different opponent cell types are not always sufficiently disambiguated. Sometimes, opponent cells within the same species and experiment have the same cone inputs but exhibit spectrally different responses (as measured by their null points) (Ventura et al., 1999; Yin et al., 2009; Djamgoz & Greenstreet, 1996; Burkhardt & Hassin, 1983).

By specifying the null points of each opponent cell type, we can disambiguate the overlapping classifications. Should we, then, just use null points as our basis for separating out cell types? Unfortunately, the null point also leaves an incomplete picture on its own. First, as discussed in ‘Experimental design elements influencing opponent cell discovery’, the null point of an opponent cell varies with light intensity (Twig, Levy & Perlman, 2003). Twig, Levy & Perlman (2003) identifies cases where the null point for the same class of cells varies between studies. This is because the null point is not a single, constant value for each cell type, it depends on the experimental conditions. Second, we again turn to the Siberian sturgeon (*Acipenser baeri*) as an example. Both R/G_1_ and R/G_2_ have a null point positioned at 600 nm, but with different photoreceptor inputs. Classifying these cells based solely on null points would not disambiguate these cell types. Therefore, the clearest opponent cell classification is based on the photoreceptor inputs and spectral properties when available. Regardless, we suggest that the null point(s) for each cell should be reported whenever known.

## Conclusions

Opponency has been studied in many species; evidence of opponency has been found in mammals, reptiles, amphibians, fish, and birds. Fish encompass the most species sampled. In the species studied so far, it seems that opponent cell types do not always persist through the retina. When comparing opponent cells from different layers of the visual system within species, we observed that the cell types reported in each species vary by layer. Yet, opponency has not been studied in more than one visual system layer in most species. While most of the cells contributing to opponency are the single cones, double cones and rods have all been found to contribute. Of mammals, reptiles, amphibians, and fish, only reptiles have shown opponent cells exclusively driven by single cones. Although the only reptiles sampled have been turtles, and these results may change when more reptile species are studied. Due to the limited reporting of sampling metrics, we have a limited ability to draw strong conclusions about the impact of sampling on opponent cell discovery. However, there are experimental design elements which are known to impact opponent cell discovery and can be controlled. Existing identifiers of opponent mechanisms are not meant for broad comparisons across species and visual system layers. We provide three broadly applicable classification systems which can be applied to any opponent cell for which photoreceptor inputs are known. These classification systems may be used to elucidate trends across opponent mechanisms going forward.

## Supplemental Information

10.7717/peerj.20959/supp-1 Supplemental Information 1 Background information on opponency (A) Additive and subtractive comparisons between photoreceptor signals. (Ai) Photoreceptor 1 is maximally sensitive in the greens, and photoreceptor 2 is maximally sensitive in the oranges. When the signals from both photoreceptors are summed together the resulting sensitivity is broader than the individual photoreceptors. The resulting signal indicates luminance without regard for specific wavelengths. The non-opponent cell reacts similarly to light, regardless of wavelength. (Aii) When the signals from photoreceptor two are subtracted from photoreceptor 1 by an opponent, the resulting sensitivity is narrower than both photoreceptors individually. The opponent cell reacts differently to light based on the wavelength. It depolarizes to some wavelengths and hyperpolarizes to others. (B) Retinal schematic showing the layers of the retina where opponent cells can be found. The top of the image displays the photoreceptors, which are the most upstream cells. Information is transmitted downstream to the retinal ganglion cells. These cells send information further downstream to other visual system layers. (C) An example of a possible opponent cell spectral response. This cell is excited by blues and yellows and inhibited by greens and reds. This cell has two peaks, labeled with circles, and two troughs labeled with hexagons. This cell has three null points, where the cell response transitions between excitation and inhibition, which are marked with diamonds. (D) The types of spectral responses that opponent cells can exhibit. Opponent cells can exhibit biphasic responses, with one excitatory phase and one inhibitory phase, and a null point in between. They can also exhibit triphasic responses (with three phases and two null points) or tetraphasic (with four phases and three null points). Table 2. We classified the opponent horizontal cells in the Siberian sturgeon (Acipenser baeri) reported by Govardovskki et al. (85) according to the three methods. Horizontal Cell Type refers to the cell classification in the original paper, with subscripts 1 and 2 added to disambiguate the subtypes of R/G cells. The null point(s) refer to the wavelength(s) between the cell’s excitatory and inhibitory responses, where it does not respond to light. The remaining three columns are how these cells would be classified according to the three methods we propose.

10.7717/peerj.20959/supp-2 Supplemental Information 2 Reproducible literature search details The search queries and a simplified PRISMA diagram illustrating our search strategy. More information can be found at doi: 10.1016/j.dib.2024.111166

10.7717/peerj.20959/supp-3 Supplemental Information 3 Supplementary methods

10.7717/peerj.20959/supp-4 Supplemental Information 4 Data used to classify opponent cells

10.7717/peerj.20959/supp-5 Supplemental Information 5 Number of species studied in each major vertebrate class and visual system layer

10.7717/peerj.20959/supp-6 Supplemental Information 6 Percent of spectrally opponent cells with biphasic, triphasic, and tetraphasic responses

10.7717/peerj.20959/supp-7 Supplemental Information 7 Photoreceptors contributing to opponency across major vertebrate classes We consider the photoreceptor cell types which are contributing to cone opponency in each of the four major vertebrate classes. A check mark (√) indicates that the above photoreceptor cell type contributes to cone opponency in at least one species belonging to the adjacent major vertebrate class. A circle with a diagonal slash indicates that the major vertebrate class has not been reported to possess that type of photoreceptor. An X indicates that the photoreceptor cell type is present but not reported to be involved in cone opponency.

10.7717/peerj.20959/supp-8 Supplemental Information 8 Spectral properties of opponent cells reported in multiple visual system layers

10.7717/peerj.20959/supp-9 Supplemental Information 9 Spatial properties of opponent cells reported in multiple visual system layers

10.7717/peerj.20959/supp-10 Supplemental Information 10 Method for classifying spatially complex opponent cells

10.7717/peerj.20959/supp-11 Supplemental Information 11 Classifying spatially complex cells Sankey plot illustrating how spatially complex opponent cells can be represented with multiple spatially simple classifications. The two columns show the spatial dimension of opponent cells. In the first column, cells are represented in their spatially complex format. The second column shows spatially simple representations. The height of the block indicates the number of cells classified this way. Grey lines indicate how the spatially complex cell (left) can be represented using multiple spatially simple classifications (right).

## Additional Information and Declarations

### Data Availability

The following information was supplied regarding data availability: The data is available at Open Science Framework: Teed, Carlay L. L. 2026. “Taxonomic Distribution, Sampling, and Classification of Opponent Cells.” OSF. January 27. doi:10.17605/OSF.IO/CM689.
