# Supplementary figures and images for: Visual opponent mechanisms and spectral responses in non-primate vertebrates: taxonomic distribution, sampling, and classification
Source: PeerJ. 2026 Mar 20;14:e20959. doi: 10.7717/peerj.20959 (PMC13007642; doi:10.7717/peerj.20959)

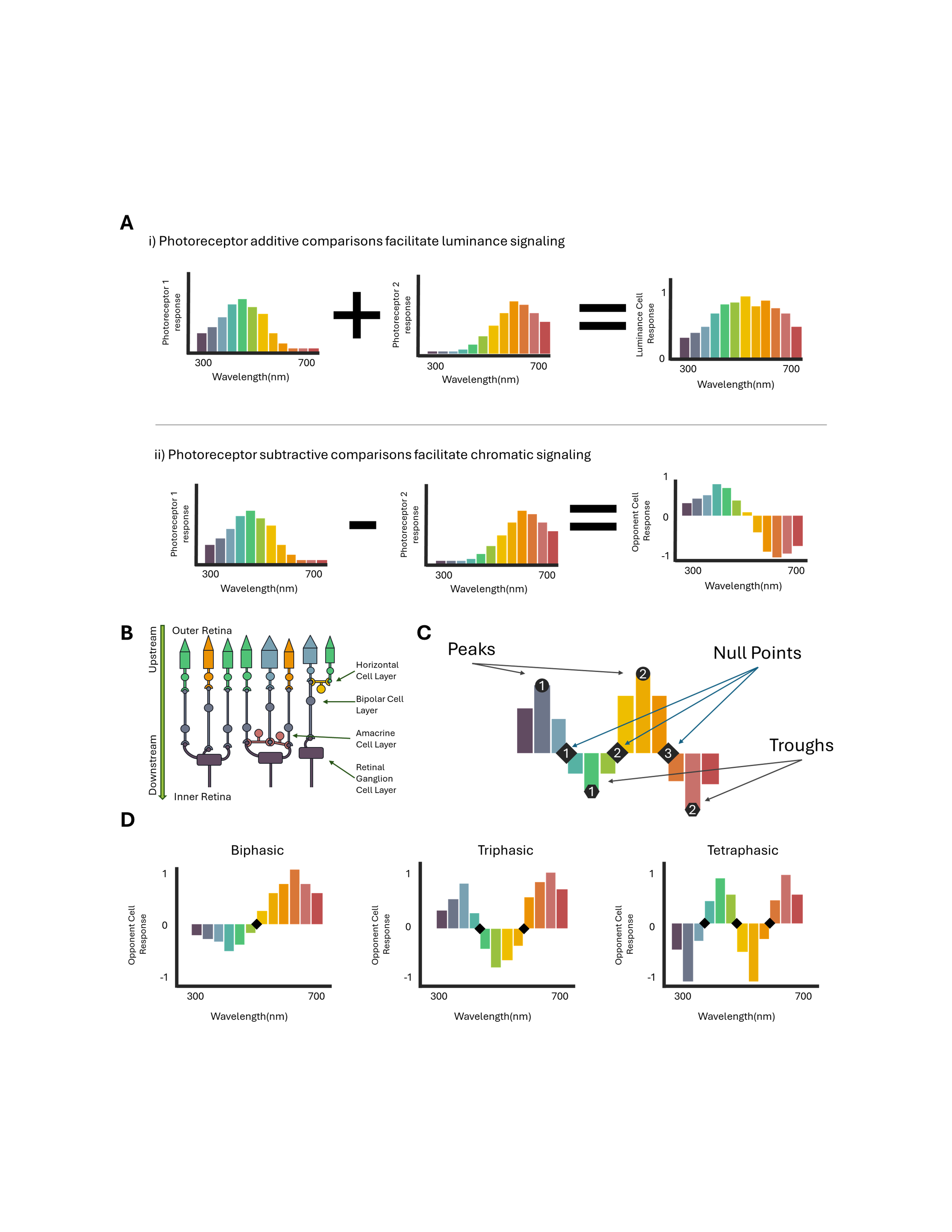

Supplement: Supplemental Information 1 — (A) Additive and subtractive comparisons between photoreceptor signals. (Ai) Photoreceptor 1 is maximally sensitive in the greens, and photoreceptor 2 is maximally sensitive in the oranges. When the signals from both photoreceptors are summed together the resulting sensitivity is broader than the individual photoreceptors. The resulting signal indicates luminance without regard for specific wavelengths. The non-opponent cell reacts similarly to light, regardless of wavelength. (Aii) When the signals from photoreceptor two are subtracted from photoreceptor 1 by an opponent, the resulting sensitivity is narrower than both photoreceptors individually. The opponent cell reacts differently to light based on the wavelength. It depolarizes to some wavelengths and hyperpolarizes to others. (B) Retinal schematic showing the layers of the retina where opponent cells can be found. The top of the image displays the photoreceptors, which are the most upstream cells. Information is transmitted downstream to the retinal ganglion cells. These cells send information further downstream to other visual system layers. (C) An example of a possible opponent cell spectral response. This cell is excited by blues and yellows and inhibited by greens and reds. This cell has two peaks, labeled with circles, and two troughs labeled with hexagons. This cell has three null points, where the cell response transitions between excitation and inhibition, which are marked with diamonds. (D) The types of spectral responses that opponent cells can exhibit. Opponent cells can exhibit biphasic responses, with one excitatory phase and one inhibitory phase, and a null point in between. They can also exhibit triphasic responses (with three phases and two null points) or tetraphasic (with four phases and three null points). Table 2. We classified the opponent horizontal cells in the Siberian sturgeon (Acipenser baeri) reported by Govardovskki et al. (85) according to the three methods. Horizontal Cell Type re [file peerj-14-20959-s001.png]

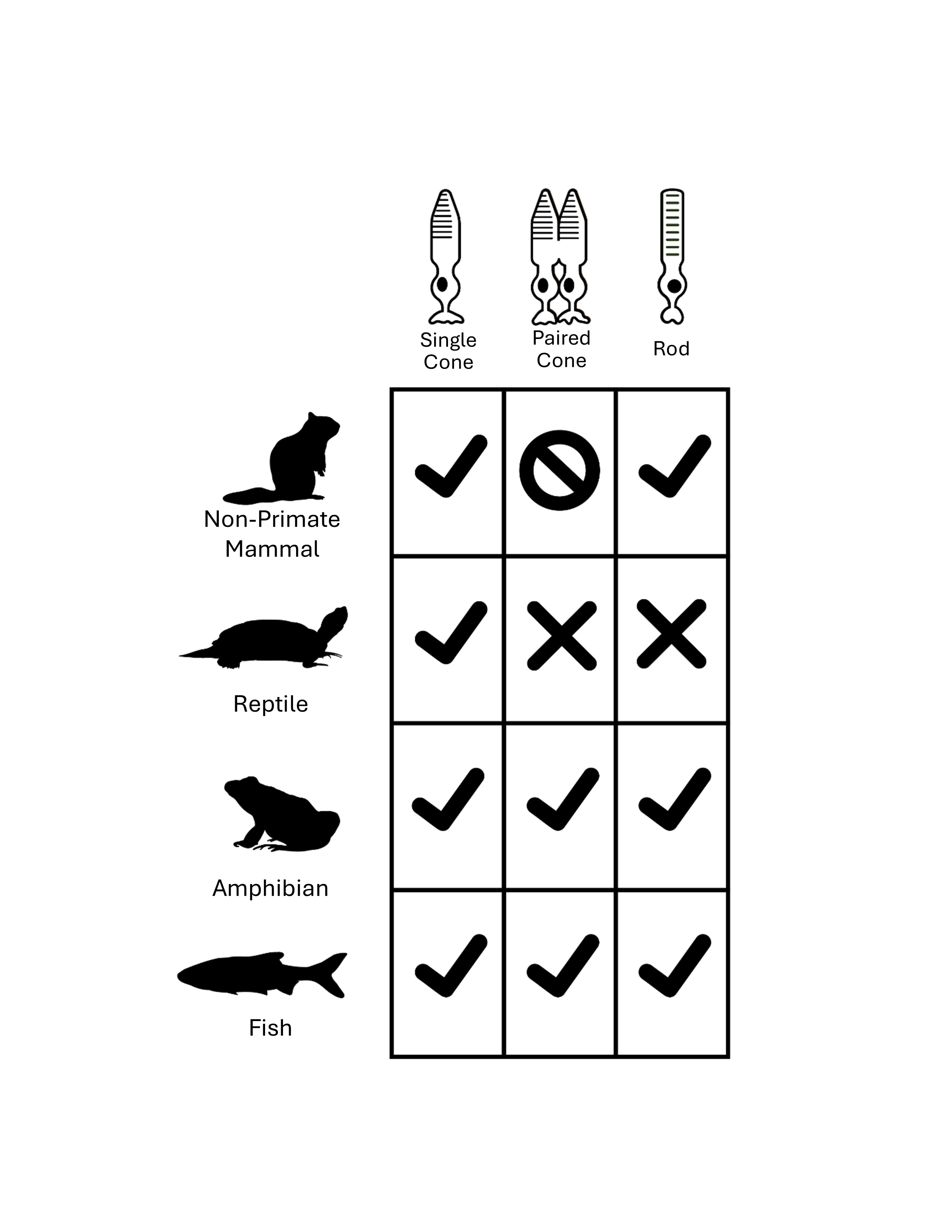

Supplement: Supplemental Information 7 — We consider the photoreceptor cell types which are contributing to cone opponency in each of the four major vertebrate classes. A check mark (√) indicates that the above photoreceptor cell type contributes to cone opponency in at least one species belonging to the adjacent major vertebrate class. A circle with a diagonal slash indicates that the major vertebrate class has not been reported to possess that type of photoreceptor. An X indicates that the photoreceptor cell type is present but not reported to be involved in cone opponency. [file peerj-14-20959-s007.png]

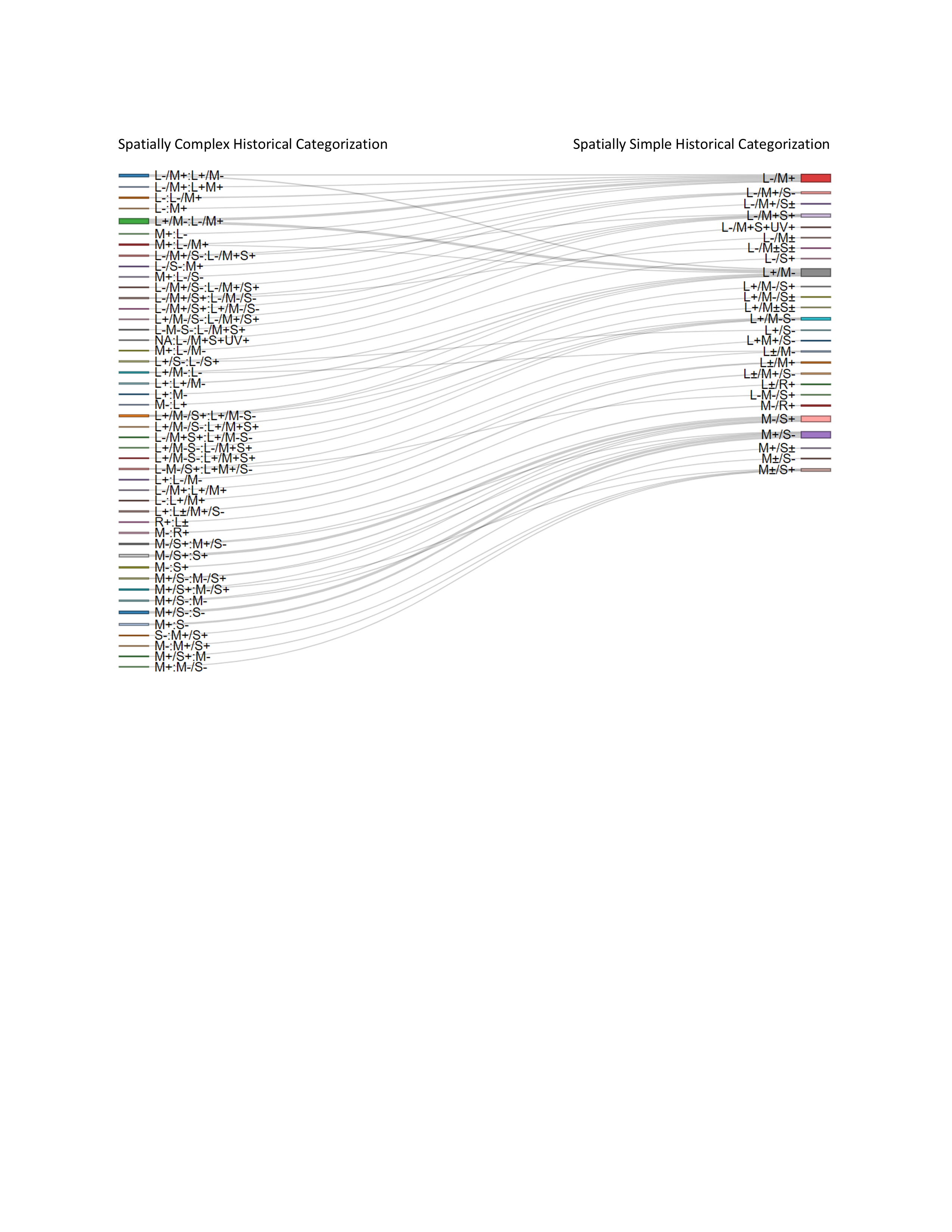

Supplement: Supplemental Information 11 — Sankey plot illustrating how spatially complex opponent cells can be represented with multiple spatially simple classifications. The two columns show the spatial dimension of opponent cells. In the first column, cells are represented in their spatially complex format. The second column shows spatially simple representations. The height of the block indicates the number of cells classified this way. Grey lines indicate how the spatially complex cell (left) can be represented using multiple spatially simple classifications (right). [file peerj-14-20959-s011.png]
