# Supplementary material for: Visual opponent mechanisms and spectral responses in non-primate vertebrates: taxonomic distribution, sampling, and classification
Source: PeerJ. 2026 Mar 20;14:e20959. doi: 10.7717/peerj.20959 (PMC13007642; doi:10.7717/peerj.20959)

Search Query:
Web of Science: “AB=((c-type OR oppon*) AND (photoreceptor OR horizontal OR bipolar OR amacrine OR ganglion OR nucleus) AND (retina OR antagonistic OR null)) OR TI =((c-type OR oppon*) AND (photoreceptor OR horizontal OR bipolar OR amacrine OR ganglion OR nucleus) AND (retina OR antagonistic OR null)) OR KP = ((c-type OR oppon*) AND (photoreceptor OR horizontal OR bipolar OR amacrine OR ganglion OR nucleus) AND (retina OR antagonistic OR null))”

Scopus: “TITLE-ABS-KEY (c-type OR oppon*) AND (photoreceptor OR horizontal OR bipolar OR amacrine OR ganglion OR nucleus) AND (retina OR antagonistic OR null) AND NOT ("Vitamin C" OR "Vit C")”

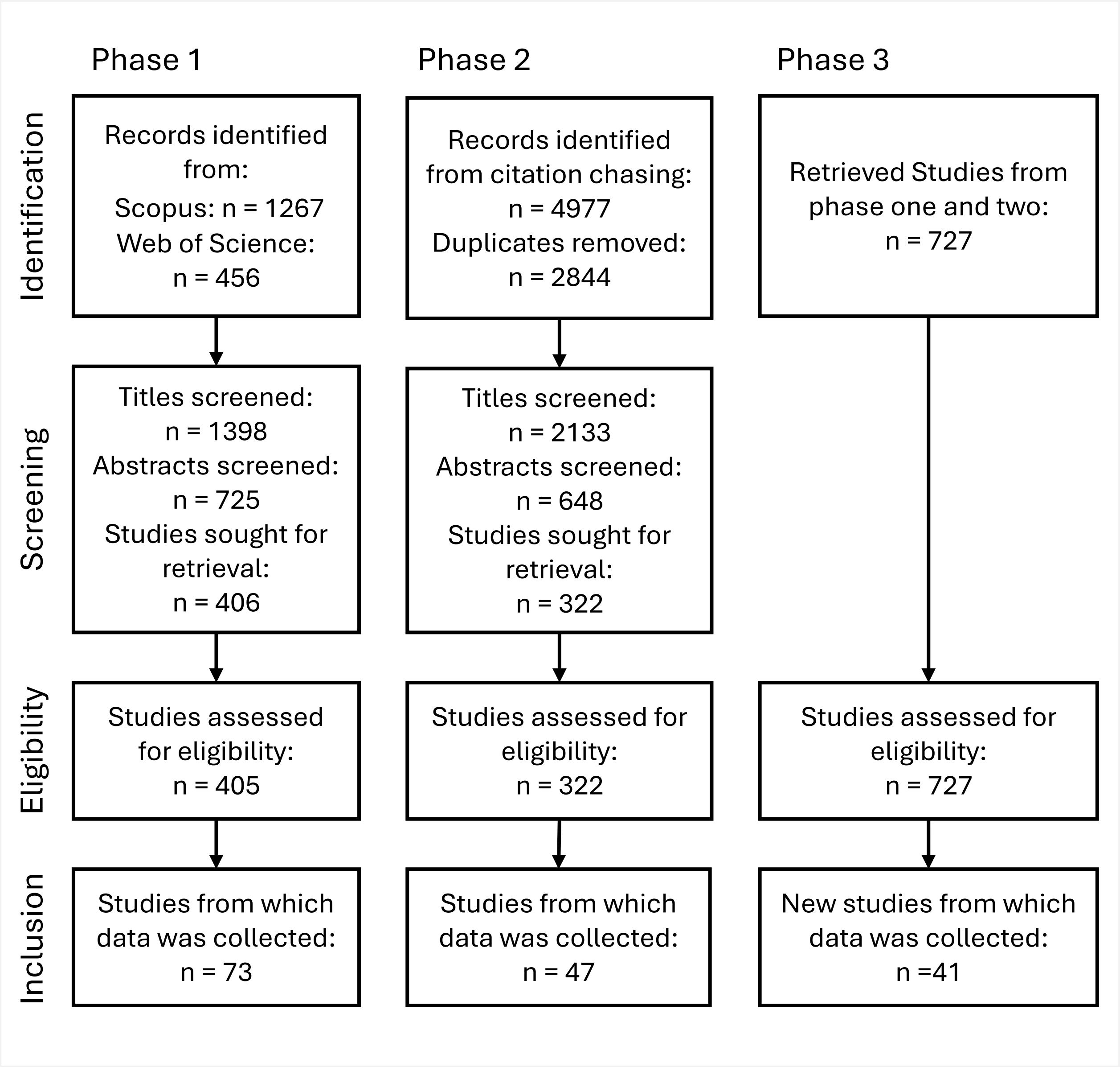

Supplement: Supplemental Information 2 — The search queries and a simplified PRISMA diagram illustrating our search strategy. More information can be found at doi: 10.1016/j.dib.2024.111166 [file peerj-14-20959-s002.pdf]
