# Supplementary material for: Visual opponent mechanisms and spectral responses in non-primate vertebrates: taxonomic distribution, sampling, and classification
Source: PeerJ. 2026 Mar 20;14:e20959. doi: 10.7717/peerj.20959 (PMC13007642; doi:10.7717/peerj.20959)
Supplement: Supplemental Information 3 [file peerj-14-20959-s003.docx]

**Supplementary Methods:**

*Distribution of opponency across species*

We extracted Taxon ID, Major vertebrate class, Genus, Species, and Brain Region from the Cone Opponency Table Uniform Receptive Field Sheet. The same information was extracted from the Cone Opponency Table CS sheet. These two sheets in the Cone Opponency Table document represent cells with different types of spatial properties (We did not consider spatial properties in this section). We extrapolated presence and absence data of cone opponency for each brain region and each species. To determine the distribution of spectral opponency across species, we extracted the columns Taxon ID and Cell.Type, then used the species Key found in the Source_List file to determine the Major Vertebrate class, Genus, and Species for each cell. We also extrapolated presence and absence data of spectral opponency for each brain region.

To determine the photoreceptors that contribute to cone opponency in each major vertebrate class, we extracted the columns Major vertebrate class, L+, L-, M+, M-, S+, S-, UV+, UV-, Rod +, Rod-, DC primary +, DC Primary-, DC Secondary+, DC Secondary- from the Cone Opponency Table Uniform Receptive Field sheet. The same information was extracted from the Cone Opponency Table CS sheet. These columns already encode cone input as presence/absence data. We compiled information on which cones provided input to any cone opponent cell in each major vertebrate class.

To examine trends in species for which more than one visual system layer was studied, we used the aforementioned dataset on distribution of cone opponency across species and extracted the number of species that were studied in more than one visual system layer. For these species, we extracted the number of opponent cell types at each layer by counting the number of rows (because each row represents one opponent cell). This allowed us to assess how the number of opponent cell types varies as one moves downstream.

We also extracted information about the spatial properties at different visual system layers. We determined the spatial properties at each visual system layer by noting which excel sheet in the Cone Opponency Table each species and each brain region is represented on (Uniform receptive field and CS). We examined if the spatial properties changed across visual system layers farther downstream. We applied a similar process for spectral opponency.

*Sampling Opponent Cells*

We extracted four sampling metrics from the Uniform receptive field and CS sheets of the Cone Opponency document. We defined sampling effort as the number of cells recorded in a given study. We extracted this from the column “# of Cells Recorded”. We define number of opponent cells found (regardless of the opponent cell classification) as the total number of opponent cells sampled in each study. This information comes from the column “# of opponent Cells Found”. We define the number of opponent cell types as the number of unique opponent cell types. We determine this value by counting the number of rows for each species after removing duplicate rows which have the same cone inputs with the same polarities. Lastly, we define the number of opponent cells in each class as the number of times a given cell type was encountered in each study. This information was extracted from the column “# of this cell”.

We calculated the percentage of opponent cells encountered in each study by dividing the number of opponent cells found by the sampling effort and multiplying the value by 100. We calculated this percentage for each unique combination of species, visual system layer, and study. Some studies have multiple percentages reported because they sampled multiple species or sampled multiple visual system layers.

We determined the percentage of studies reporting each sampling metric by first determining the total number of studies. Next, for each sampling metric, we counted all studies which included a numeric value in its respective column (e.g. number of studies reporting sampling effort is the number of cells with a numeric value in the column “# of Cells Recorded”). To calculate the percentage, we divided the number of studies reporting each sampling metric by the total number of studies and multiplied the value by 100. We determined the percentage of studies which reported all sampling metrics by determining the number of studies which reported all sampling metrics, divided that value by the total number of studies, and multiplied it by 100.

We produced a histogram of the sampling effort applied in each study by extracting the sampling effort from the column “# of Cells Recorded”, with each study represented one type per species and/or one time per visual system layer. This is because the same study may report multiple sampling efforts if different species or different visual system layers are studied at one time. We then produced a histogram in R using those values.

*Opponent Cell Classifications*

We assigned each cell a functional descriptor based on the three methods outlined in section V. We constructed two Sankey plots (Supplementary figures 1 and 2) to demonstrate how each cell may be classified in multiple ways. In the first Sankey plot, each opponent cell reported is represented one time in each column. In the second Sanky plot, only spatially complex cells are represented. This plot shows the spatially complex classifications on the left and the spatially simple transformations on the right. The same spatially complex opponent cell can be represented as one or two spatially simple representations.
