# Supplementary material for: Visual opponent mechanisms and spectral responses in non-primate vertebrates: taxonomic distribution, sampling, and classification
Source: PeerJ. 2026 Mar 20;14:e20959. doi: 10.7717/peerj.20959 (PMC13007642; doi:10.7717/peerj.20959)
Supplement: Supplemental Information 5 [file peerj-14-20959-s005.docx]

| Opponency Type | Major Vertebrate Class | # of Species Studied | Visual system layer | # of Species Studied |
| --- | --- | --- | --- | --- |
| Cone Opponency | Mammal | 7 | Retinal Ganglion Cells | 7 |
|  |  |  | Lateral Geniculate Nucleus | 2 |
|  |  |  | Striate Cortex | 1 |
|  | Reptile | 3 | Horizontal Cells | 3 |
|  |  |  | Bipolar Cells | 2 |
|  |  |  | Retinal Ganglion Cells | 1 |
|  | Amphibian | 5 | Horizontal Cells | 2 |
|  |  |  | Bipolar Cells | 1 |
|  |  |  | Retinal Ganglion Cells | 3 |
|  | Fish | 16 | Horizontal Cells | 11 |
|  |  |  | Bipolar Cells | 3 |
|  |  |  | Amacrine Cells | 3 |
|  |  |  | Retinal Ganglion Cells | 2 |
|  |  |  | Optic Tectum | 3 |
|  |  |  | Torus Semicircularus | 1 |
| Spectral Opponency | Mammal | 5 | Retinal Ganglion Cells | 3 |
|  |  |  | Lateral Geniculate Nucleus | 5 |
|  | Reptile | 3 | Horizontal Cells | 3 |
|  |  |  | Bipolar Cells | 1 |
|  |  |  | Amacrine Cells | 1 |
|  |  |  | Retinal Ganglion Cells | 1 |
|  | Amphibian | 6 | Horizontal Cells | 2 |
|  |  |  | Bipolar Cells | 1 |
|  |  |  | Retinal Ganglion Cells | 3 |
|  |  |  | Optic Tectum | 2 |
|  | Fish | 44 | Horizontal Cells | 41 |
|  |  |  | Bipolar Cells | 5 |
|  |  |  | Amacrine Cells | 5 |
|  |  |  | Retinal Ganglion Cells | 4 |
|  |  |  | Optic Tectum | 1 |
|  |  |  | Torus Semicircularis | 1 |
