# Supplementary material for: Visual opponent mechanisms and spectral responses in non-primate vertebrates: taxonomic distribution, sampling, and classification
Source: PeerJ. 2026 Mar 20;14:e20959. doi: 10.7717/peerj.20959 (PMC13007642; doi:10.7717/peerj.20959)
Supplement: Supplemental Information 6 [file peerj-14-20959-s006.docx]

| Major Vertebrate Class | Brain Region | Percent Biphasic | Percent Triphasic | Percent Tetraphasic |
| --- | --- | --- | --- | --- |
| Reptile | Horizontal Cells | 90% | 10% | 0% |
|  | Bipolar Cells | 100% | 0% | 0% |
|  | Amacrine Cells | 100% | 0% | 0% |
|  | Retinal Ganglion Cells | 44.4̅% | 33.3̅% | 22.2̅% |
| Mammal | Retinal Ganglion Cells | 100% | 0% | 0% |
|  | Lateral Geniculate Nucleus | 100% | 0% | 0% |
| Amphibian | Horizontal Cells | 100% | 0% | 0% |
|  | Bipolar Cells | 100% | 0% | 0% |
|  | Retinal Ganglion Cells | 66.6̅% | 33.3̅% | 0% |
|  | Optic Tectum | 66.6̅% | 33.3̅% | 0% |
| Fish | Horizontal Cells | 60.2% | 36.7% | 3% |
|  | Bipolar Cells | 84.6% | 15.4% | 0% |
|  | Amacrine Cells | 71.4% | 14.3% | 14.3% |
|  | Retinal Ganglion Cells | 78.6% | 21.4% | 0% |
|  | Optic Tectum | 66.6̅% | 33.3̅% | 0% |
|  | Torus Semicircularus | 50% | 50% | 0% |

Supplementary Table 2: Percent of Spectrally Opponent Cells with Biphasic, Triphasic, and Tetraphasic responses
