# Supplementary material for: Visual opponent mechanisms and spectral responses in non-primate vertebrates: taxonomic distribution, sampling, and classification
Source: PeerJ. 2026 Mar 20;14:e20959. doi: 10.7717/peerj.20959 (PMC13007642; doi:10.7717/peerj.20959)
Supplement: Supplemental Information 8 [file peerj-14-20959-s008.docx]

|  | Horizontal | Bipolar | Amacrine | Retinal Ganglion | Optic Tectum | Lateral Geniculate | Torus Semi-circularis |
| --- | --- | --- | --- | --- | --- | --- | --- |
| Red-eared slider (*Trachemys scripta elegans*) | Biphasic, Triphasic | Biphasic |  | Biphasic, Triphasic, Tetraphasic | Biphasic, Triphasic |  | Biphasic, Triphasic |
| California ground squirrel (*Otospermophilius beecheyi*) |  |  |  | Biphasic |  | Biphasic |  |
| Cat (*Felis catus*) |  |  |  | Biphasic |  | Biphasic |  |
| African clawed frog (*Xenopus laevis*) | Biphasic | Biphasic |  |  |  |  |  |
| Edible frog (*Pelophylax esculentus*) |  |  |  | Triphasic | Triphasic |  |  |
| Northern leopard frog (*Lithobates pipiens*) | Biphasic |  |  | Biphasic |  |  |  |
| Japanese Dace (*Tribolodon hakonensis*) | Biphasic, Triphasic, Tetraphasic | Biphasic | Biphasic |  |  |  |  |
| Zebrafish (*Danio rerio*) | Biphasic, Triphasic, Tetraphasic |  | Biphasic, Tetraphasic | Biphasic |  |  |  |
| Common Carp (*Cyprinus carpio)* | Biphasic, Triphasic | Biphasic, Triphasic | Triphasic | Biphasic |  |  |  |
| Crucian Carp (*Carassius carassius*) | Biphasic, Triphasic | Biphasic | Biphasic |  |  |  |  |
| Goldfish (*Carassius auratus*) | Biphasic, Triphasic | Biphasic, Triphasic | Triphasic | Biphasic |  |  |  |
| Rainbow trout (*Oncorhynchus mykiss*) | Biphasic |  |  | Biphasic, Triphasic | Biphasic, Triphasic |  | Biphasic, Triphasic |
