# Supplementary material for: Visual opponent mechanisms and spectral responses in non-primate vertebrates: taxonomic distribution, sampling, and classification
Source: PeerJ. 2026 Mar 20;14:e20959. doi: 10.7717/peerj.20959 (PMC13007642; doi:10.7717/peerj.20959)
Supplement: Supplemental Information 9 [file peerj-14-20959-s009.docx]

|  | Horizontal | Bipolar | Amacrine | Retinal Ganglion | Lateral Geniculate |
| --- | --- | --- | --- | --- | --- |
| Red-eared slider (*Trachemys scripta elegans*) | Simple | Simple, Complex | Simple, Complex | Simple, Complex |  |
| D’Orbigny’s slider (*Trachemys scripta elegans*) | Simple | Complex |  |  |  |
| Mexican ground squirrel (*Ictidomys mexicanus*) |  |  |  | Simple, Complex | Simple, Complex |
| California ground squirrel (*Otospermophilius beecheyi*) |  |  |  | Simple | Simple |
| Cat (*Felis catus*) |  |  |  | Complex | Complex |
| African clawed frog (*Xenopus laevis*) | Simple | Simple |  | Simple, Complex |  |
| Northern leopard frog (*Lithobates pipiens*) | Simple |  |  | Simple |  |
| Japanese Dace (*Tribolodon hakonensis*) | Simple | Complex |  | Complex |  |
| Zebrafish (*Danio rerio*) | Simple |  | Simple |  |  |
| Common Carp (*Cyprinus carpio)* | Simple | Complex |  | Complex |  |
| Crucian Carp (*Carassius carassius*) | Simple | Simple, Complex |  | Simple, Complex |  |
| Goldfish (*Carassius auratus*) | Simple | Simple |  | Simple, Complex |  |
