# Supplementary material for: Visual opponent mechanisms and spectral responses in non-primate vertebrates: taxonomic distribution, sampling, and classification
Source: PeerJ. 2026 Mar 20;14:e20959. doi: 10.7717/peerj.20959 (PMC13007642; doi:10.7717/peerj.20959)
Supplement: Supplemental Information 10 [file peerj-14-20959-s010.docx]

**Spatially complex opponent cells**

One limitation of the classification methods discussed thus far is that they do not account for spatial complexity. Twenty-seven percent of opponent cells with photoreceptor inputs reported are spatially complex and require additional processing. Here, we address how different types of spatially complex opponent cells may be classified using these three methods. Furthermore, we suggest ways to make spatially simple and complex cells comparable.

**(a) Classifying spatially complex opponent cells**

We observed three types of spatially complex opponent cells reported:

1) cells that are opponent in one portion of the receptive field, but not in the other. This can be an opponent/luminance type of opponent cell, such as L+/M- center (opponent) and L+M+ surround (non-opponent) or a cell which depolarizes to red and hyperpolarizes to green in the center, but depolarizes to both red and green in the surround;

2) cells that are non-opponent in the center and surround, but opponency emerges when the inputs to each portion of the receptive field are compared. These are single-opponent cells, such as L+ center and M- surround or a cell which depolarizes to red in the center and hyperpolarizes to green in the surround;

3) cells that are opponent in the center and surround, and the opponency in each portion of the receptive field are opposite to each other. These are double opponent cells, such as L+/M- center and M+/L- surround or a cell which depolarizes to red and hyperpolarizes to green in the center, but exhibits the opposite response in the surround.

To signify spatial complexity in our classification methods, we use a colon (:) to separate the center response from the surround response. (Daw (5) uses C: for center, and P: for periphery, but we elected to use fewer characters and drop the C and P designations). Therefore, applying the historical classification method, the opponent/luminance cell is represented as L+/M-:L+M+; the single opponent cell as L+:M-; and the double opponent cell as L+/M-:L-/M+. Applying the photoreceptor input polarity method, the opponent/luminance cell is +/-:++; the single opponent cell as +:-; and the double opponent cell as +/-:-/+. Lastly, applying the relative polarity method, the opponent/luminance cell becomes +/-:+; the single opponent cell as +:-; and the double opponent cell as +/-:-/+.

These same processes can be applied to opponent cells for which spectral responses are known but cone responses are not. We can only classify these cells according to the relative polarity method, where the opponent/luminance cell becomes +/-:+; the single opponent cell as +:-; and the double opponent cell as +/-:-/+.

**(b) Comparing spatially simple and spatially complex opponent cells**

There are occasions where one might want to compare spatially simple and complex opponent cells. To make the 27% of opponent cells that are spatially complex comparable to the spatially simple opponent cells, we can either convert spatially uniform cells into spatially complex representations or we can split the regions of the spatially complex cells into distinct spatially simple representations.

Assigning a spatially complex label to a spatially simple cell is a simple task. A spatially simple cell can be thought of as a spatially complex cell with the same properties in the center and the surround. First, the opponent cells are classified according to whichever categorization method is appropriate for the research question. Then, the same classification as assigned to the center and surround, separated by a colon (:). For example, the spatially simple *Xenopus laevis* bipolar cell L+/R- can be represented as L+/R-:L+/R-. This enables this spatially simple cell to be represented in terms which are comparable to spatially complex cells.

Assigning spatially simple labels to spatially complex cells is more complicated, owing to the three types of spatially complex opponent cells. In the following examples, we use the historical classification method, but the same process can be applied with the other two methods. After classifying each spatially complex cell, the cells’ classifications are decomposed into three elements: one part representing the center, one part representing the surround, and one combining the responses of both center and surround. Lastly, the non-opponent elements are discarded. This method results in a different number of spatially simple classifications for the three types of spatially complex cells.

For the opponent/luminance cell in our example, the L+M+ component from the surround is non-opponent and can be disregarded. We can now represent the single spatially complex cell as two spatially agnostic descriptors: L+/M- and L+/M±. A single opponent cell, L+:M-, will be broken into L+, M-, and L+/M-. The L+ and M- components are disregarded resulting in only one chromatic representation for this cell. Lastly, the double opponent cell is broken into L+/M-, L-/M+, and L±M±. The last representation is non-opponent and can be disregarded. By following this method, 45 spatially complex cells can be categorized into 26 spatially agnostic categories in the historical method (Supplementary Fig 4). Thirteen of these categories represent a single cone opponent cell, and L+/M- and L-/M+ each represent 12 cone opponent cells.
